# Supplementary material for: Novel PEI-aldehyde conjugates for gene delivery: Promoting chondrogenic differentiation in human mesenchymal stem cells
Source: Mol Ther Nucleic Acids. 2025 Apr 29;36(2):102551. doi: 10.1016/j.omtn.2025.102551 (PMC12141052; doi:10.1016/j.omtn.2025.102551)
Supplement: Document S1. Figures S1–S19 [file mmc1.pdf]

OMTN, Volume *36*

Supplemental information

Novel PEI-aldehyde conjugates for gene delivery: Promoting chondrogenic differentiation in human mesenchymal stem cells

Diego Miranda-Balbuena, Alba Ramil-Bouzas, Naiara Doldán-Mata, Junquera López-Seijas, Juan Fafián-Labora, Ibán Lamas-Criado, Jose-Ramón Caeiro-Rey, Paco Fernández-Trillo, and Ana Rey-Rico

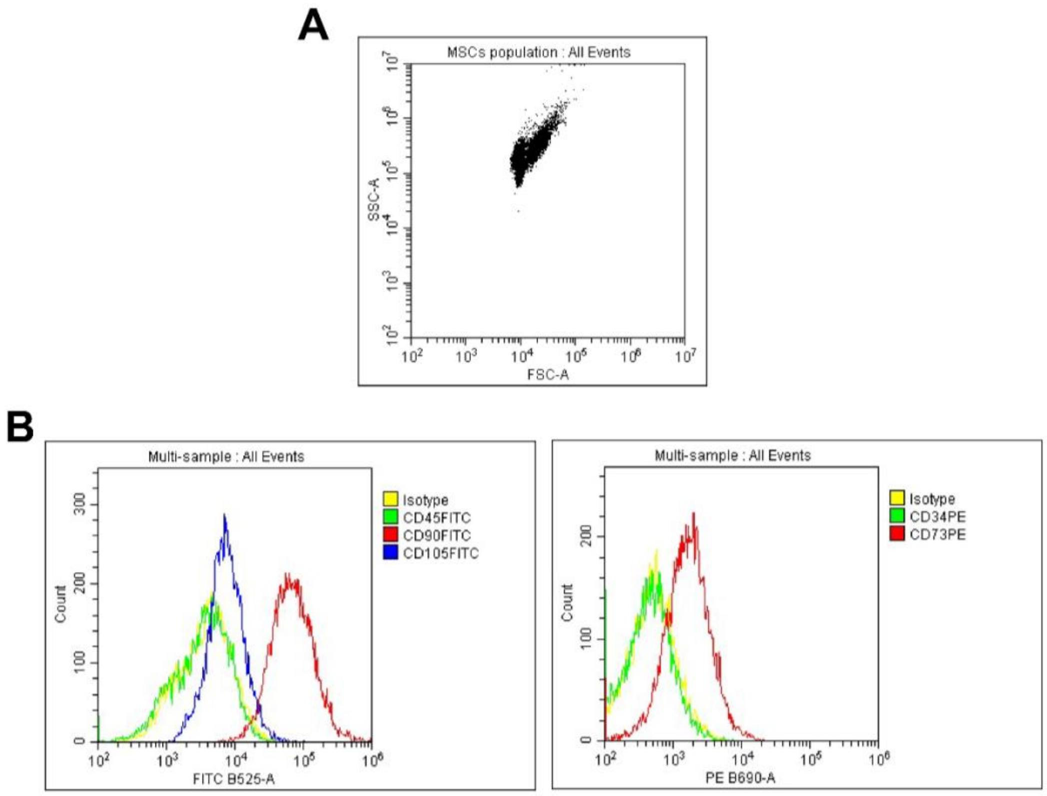

***Figure S1. Representative images for the analysis of hMSCs surface markers**. Flow cytometry analysis of hMSCs markers CD90, CD73, and CD105 and hematopoietic markers CD45, and CD34, compared with their corresponding isotype FITC or PE as negative control.*

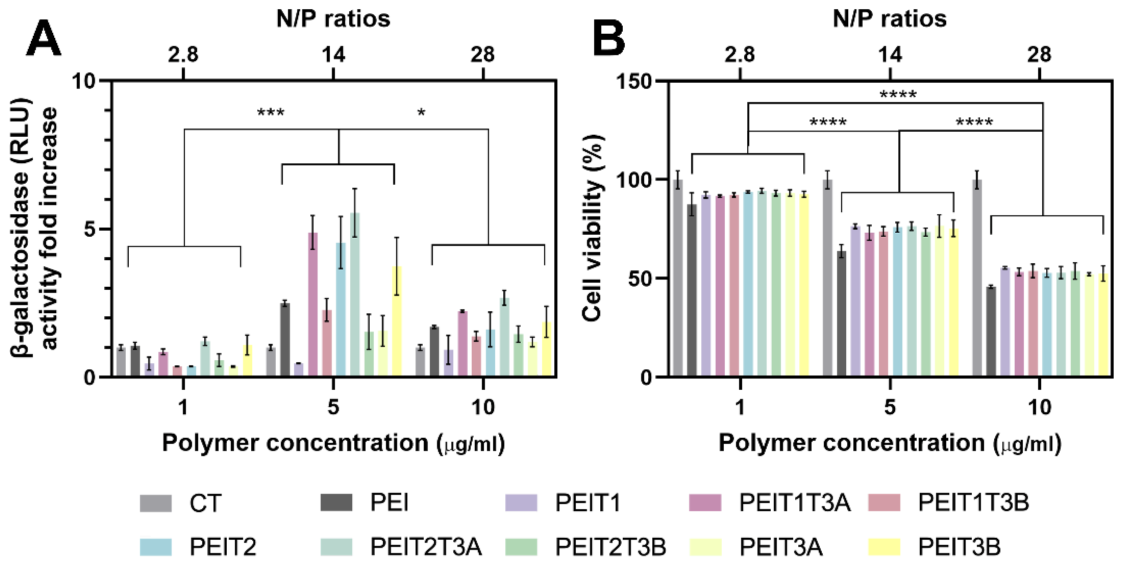

***Figure S2. Effect of broad transfectant concentrations on transfection efficiency and cell viability.** (A) β-galactosidase activity and (B) cell viability after transfection with different polyplexes (PEI, PEIT1, PEIT1T3A, PEIT1T3B, PEIT2, PEIT2T3A, PEIT2T3B, PEIT3A, and PEIT3B) (placZ) at various concentrations (1 μg/ml, 5 μg/ml, and 10 μg/ml) and N/P ratios (2.8, 14, and 28) in hMSCs. Cationic/hydrophobic aldehyde ratio = 70/30, and [placZ] = 2.5 μg/mL. * depicts $p < 0.05$, *** $p < 0.001$, and **** $p < 0.0001$ when compared denoted groups. Data is expressed as mean of quadruplicates; error bars indicate standard deviation.*

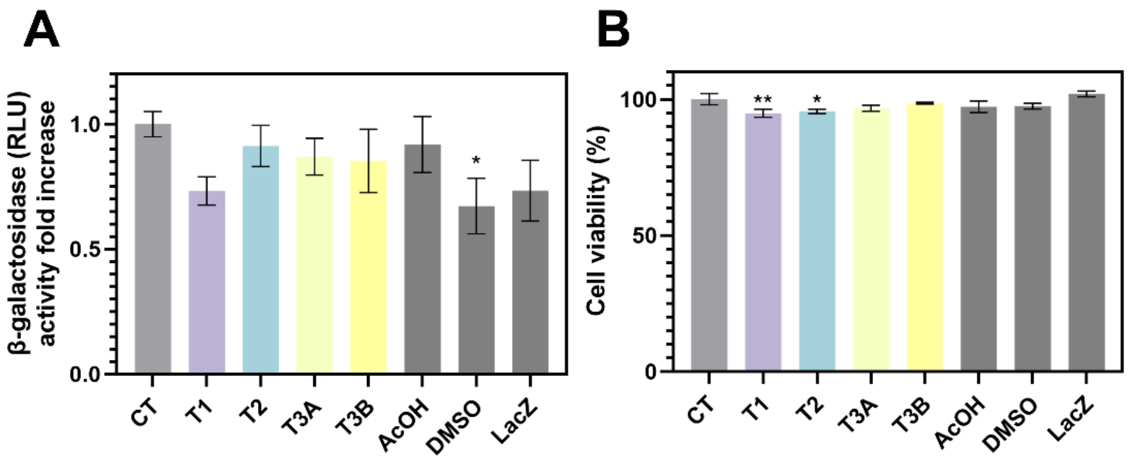

Figure S3. Transfection efficiency and cell viability of negative controls. *(A) β-galactosidase activity and (B) cell viability after transfection with different control conditions (aldehydes: T1, T2, T3A, and T3B, acetic acid (AcOH), DMSO, and plasmid placZ alone) in hMSCs. [placZ] = 2.5 µg/ml. * depicts p < 0.05, and ** p < 0.01 when compared denoted groups. Data is expressed as mean of quadruplicates; error bars indicate standard deviation.*

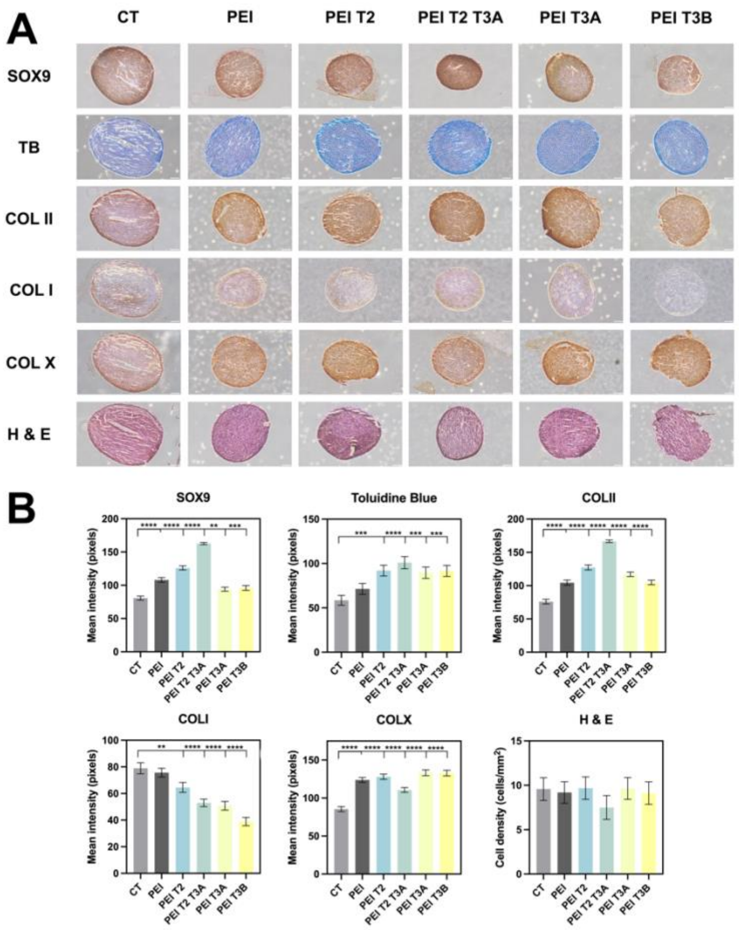

Figure S4. Immunohistochemical and histological analyses of chondrogenesis for all conditions. *Immunohistochemical and histological analyses of hMSC aggregates cultured in chondrogenic medium (CT; negative control) and transfected with psox9 via PEI, PEIT2, PEIT2T3E, PÈIT3E, or PEIT3F. Samples were kept in culture for 21 days and processed for (A) Immunodetection of SOX9, toluidine blue (TB), type-II collagen (COLII), type-I collagen (COLI), type-X collagen (COLX), and Hematoxylin/Eosin (H&E) (all representative images; magnification 10X; scale bar 100 µm). (B) Histomorphometric analyses of the previously mentioned conditions. Cationic/hydrophobic aldehyde ratio = 70/30, [PEI] = 5 µg/ml, and [placZ]*

= 5 µg/ml. ** depicts $p < 0.01$, *** $p < 0.001$, and **** $p < 0.0001$ when compared with denoted groups. Data is expressed as mean of triplicates; error bars indicate standard deviation.

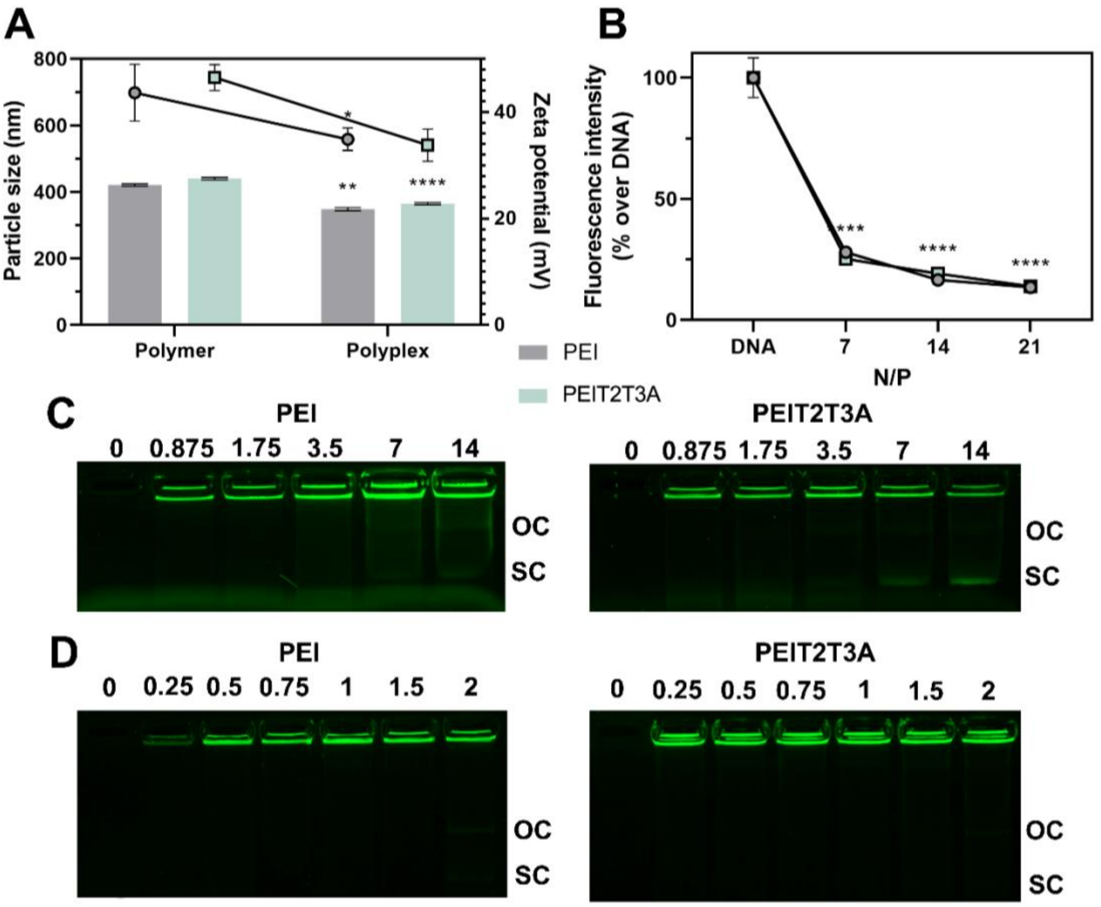

Figure S5. Unmodified PEI and PEIT2T3A characterization. *(A) Particle size (bars) and zeta potential (dots) of PEI and PEIT2T3E polymers and polyplexes (placZ). Cationic/hydrophobic aldehyde ratio = 70/30, [PEI] = 5 µg/ml, and [placZ] = 5 µg/ml (B) DNA placZ complexation efficiency of PEI and PEIT2T3E polyplexes formed at different concentrations. Cationic/hydrophobic aldehyde ratio = 70/30 and [PEI] = 5 µg/ml. (C) DNase protection ability and SDS-induced release visualized by agarose electrophoresis of PEI and PEIT2T3E formed at N/P ratios of 0.875/1, 1.75/1, 3.5/1, 7/1, and 14/1 (placZ). (D) DNase protection ability and SDS-induced release visualized by agarose electrophoresis of PEI and PEIT2T3E formed at N/P ratios of 0.25/1, 0.5/1, 0.75/1, 1/1, 1.5/1, and 2/1 (placZ). Cationic/hydrophobic aldehyde ratio = 70/30 and [PEI] = 5 µg/ml. 0: naked placZ; OC: open circular; SC: supercoiled * depicts $p < 0.05$, ** $p < 0.01$, *** $p < 0.001$, and **** $p < 0.0001$. Data is expressed as mean of quadruplicates; error bars indicate standard deviation.*

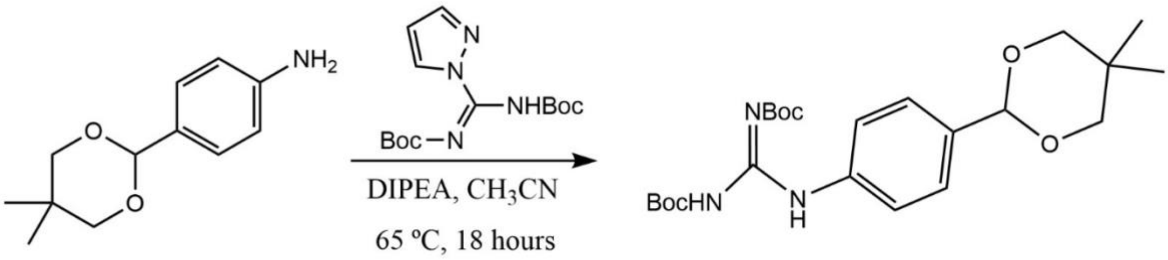

Figure S6. Synthesis of 1-(4-formylphenyl)guanidine) (T2). *Synthetic strategy for the synthesis of 1-(4-formylphenyl)guanidine) (T2).*

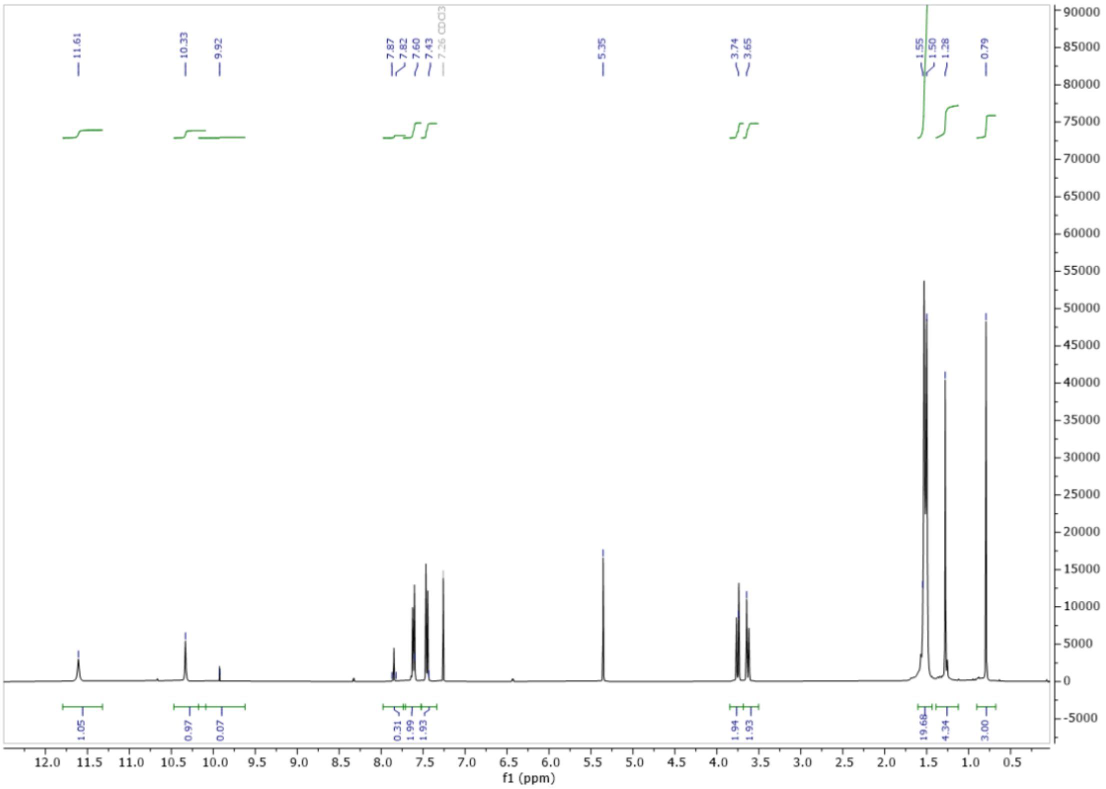

Figure S7. Synthesis of 1-(4-formylphenyl)guanidine) (T2). *Characterization of 1-(4-formylphenyl)guanidine) (T2) by H NMR (400 MHz, CDCl3).*

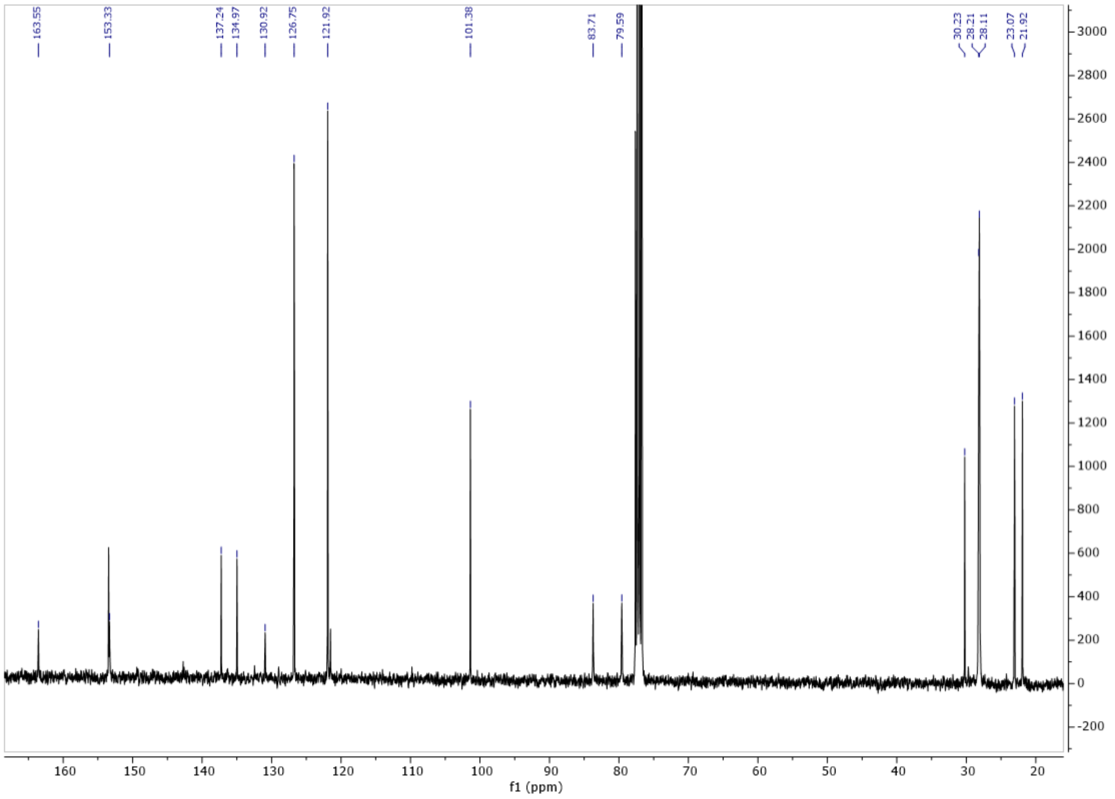

Figure S8. Synthesis of 1-(4-formylphenyl)guanidine) (T2). *Characterization of 1-(4-formylphenyl)guanidine) (T2) by C NMR (101 MHz, CDCl3).*

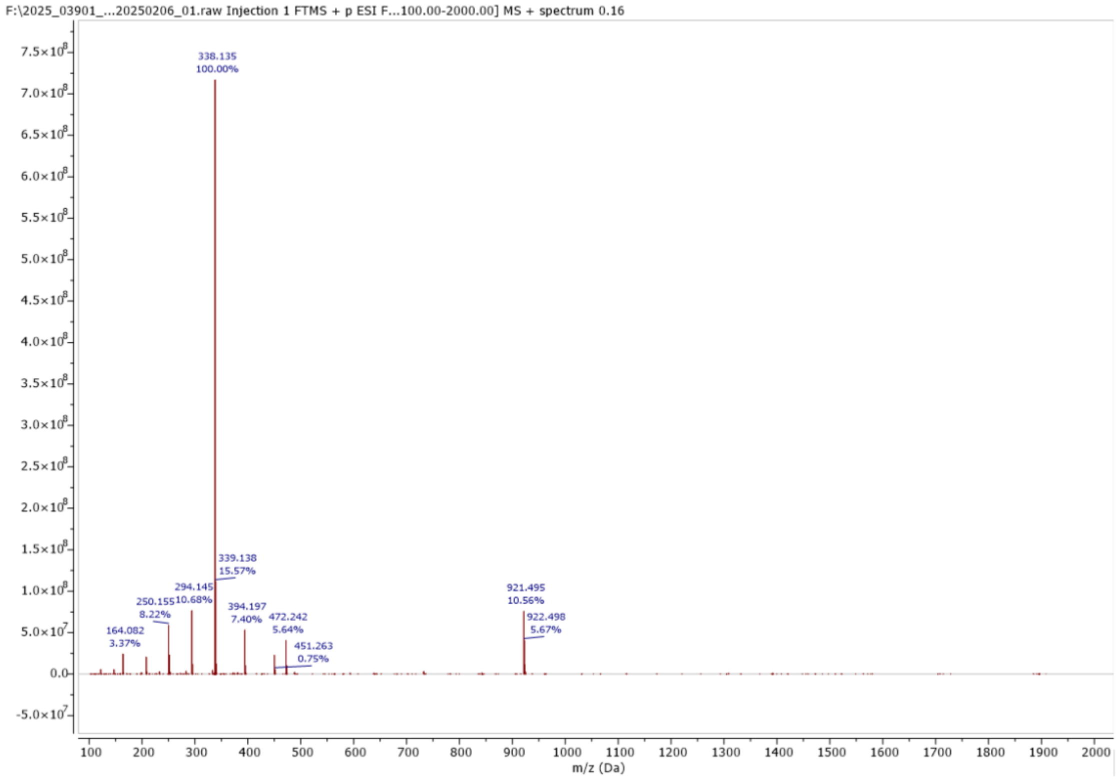

Figure S9. Synthesis of 1-(4-formylphenyl)guanidine) (T2). *Characterization of 1-(4-formylphenyl)guanidine) (T2) by ESI-MS (CH3Cl) m/z [2M+ Na]+ 921,495.*

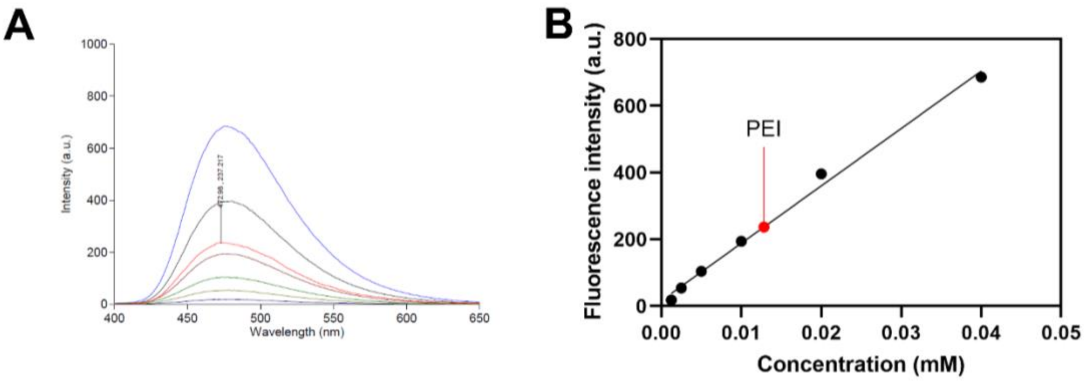

Figure S10. Estimation of unmodified PEI free amines. *Fluorescence intensity of PEI (Red line (A) or dot (B)) concerning ethanolamine (rest of the values) after functionalization of the free amines in both compounds with fluorescamine. Data is represented concerning wavelength (A) or concentration (B).*

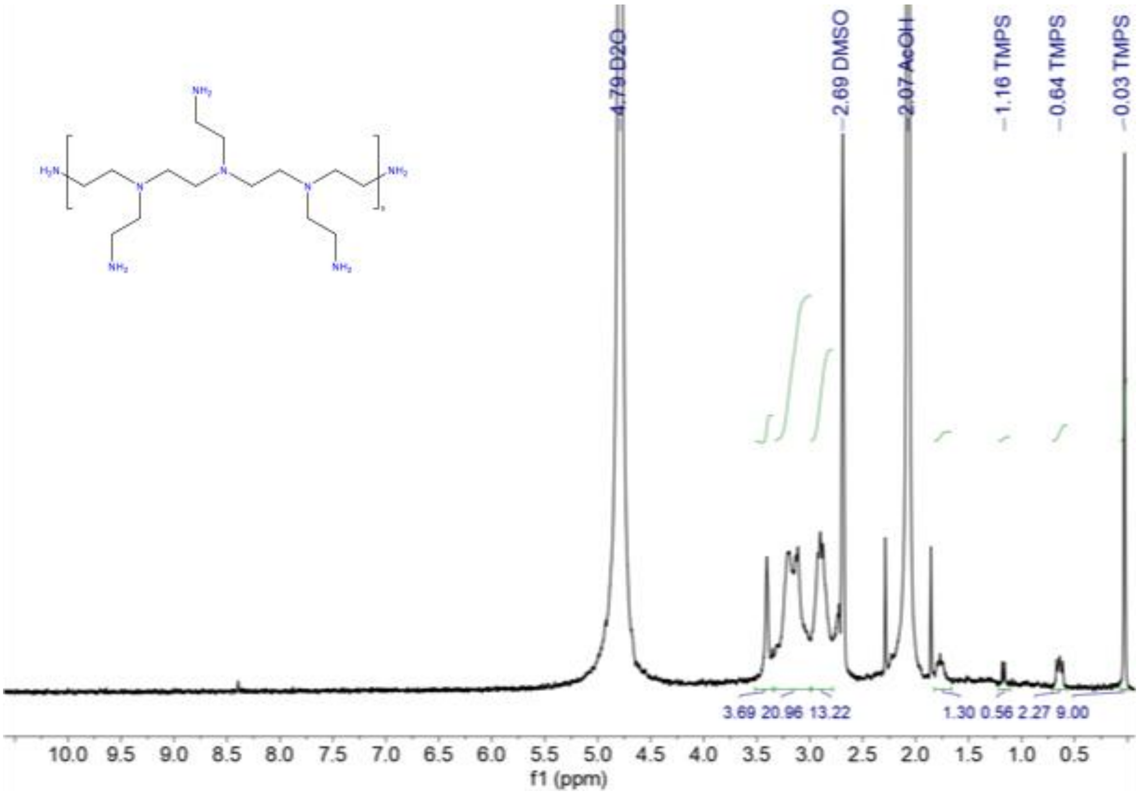

Figure S11. ^{1}H NMR spectra of PEI *in acetate buffer (100 mM, pH 3.0) in D_2O. Sodium 3-(trimethylsilyl)propane-1-sulfonate (**TMPS**) used as an internal reference.*

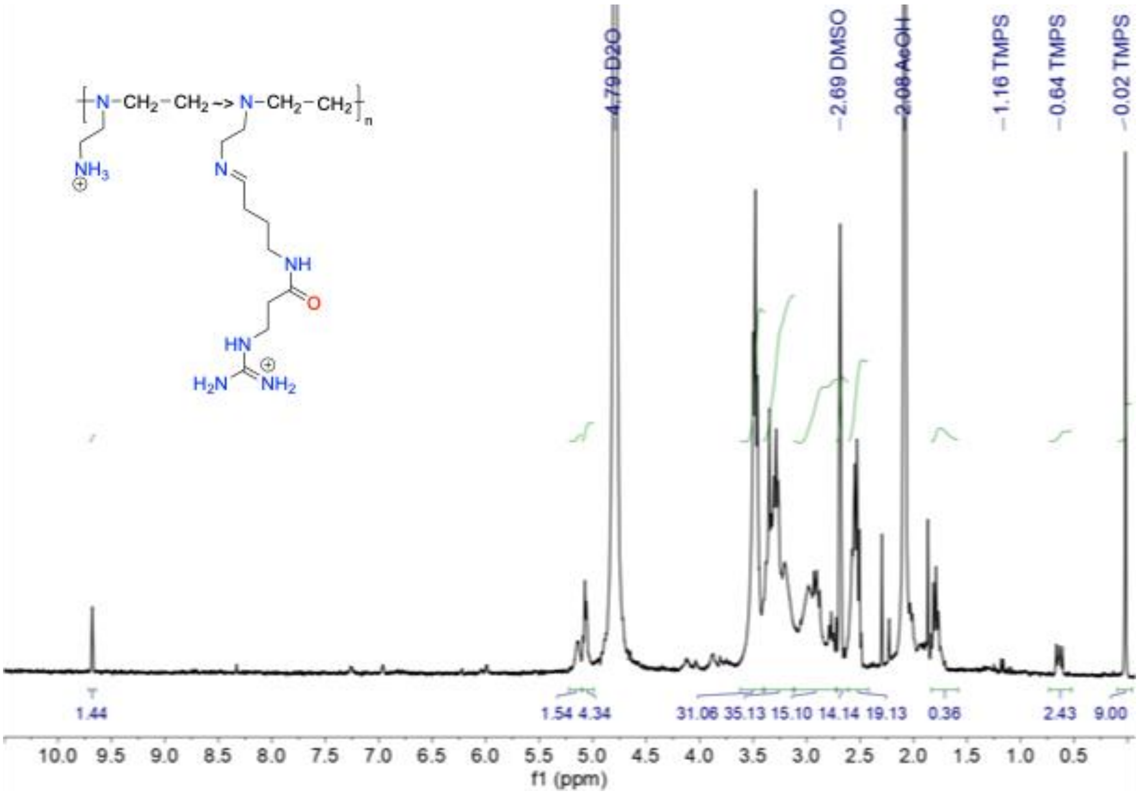

Figure S12. ^{1}H NMR spectra of p[PEI-mod-T1$_{0.30}$] (PEIT1) *in acetate buffer (100 mM, pH 3.0) in D_2O. Sodium 3-(trimethylsilyl)propane-1-sulfonate (**TMPS**) used as an internal reference.*

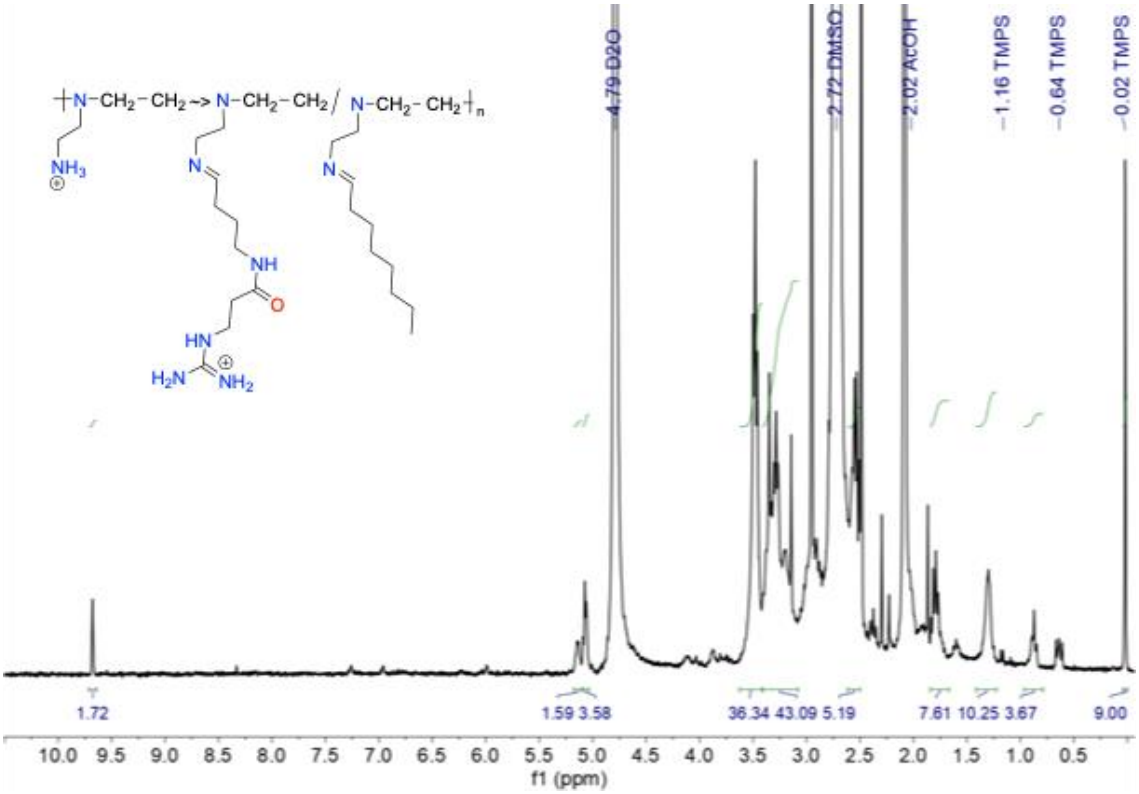

***Figure S13.** **^{1}H NMR spectra of p[PEI-mod-T1$_{0.32}$T3A$_{0.6}$] (PEIT1T3A)** in acetate buffer (100 mM, pH 3.0) in D_2O. Sodium 3-(trimethylsilyl)propane-1-sulfonate (**TMPS**) used as an internal reference.*

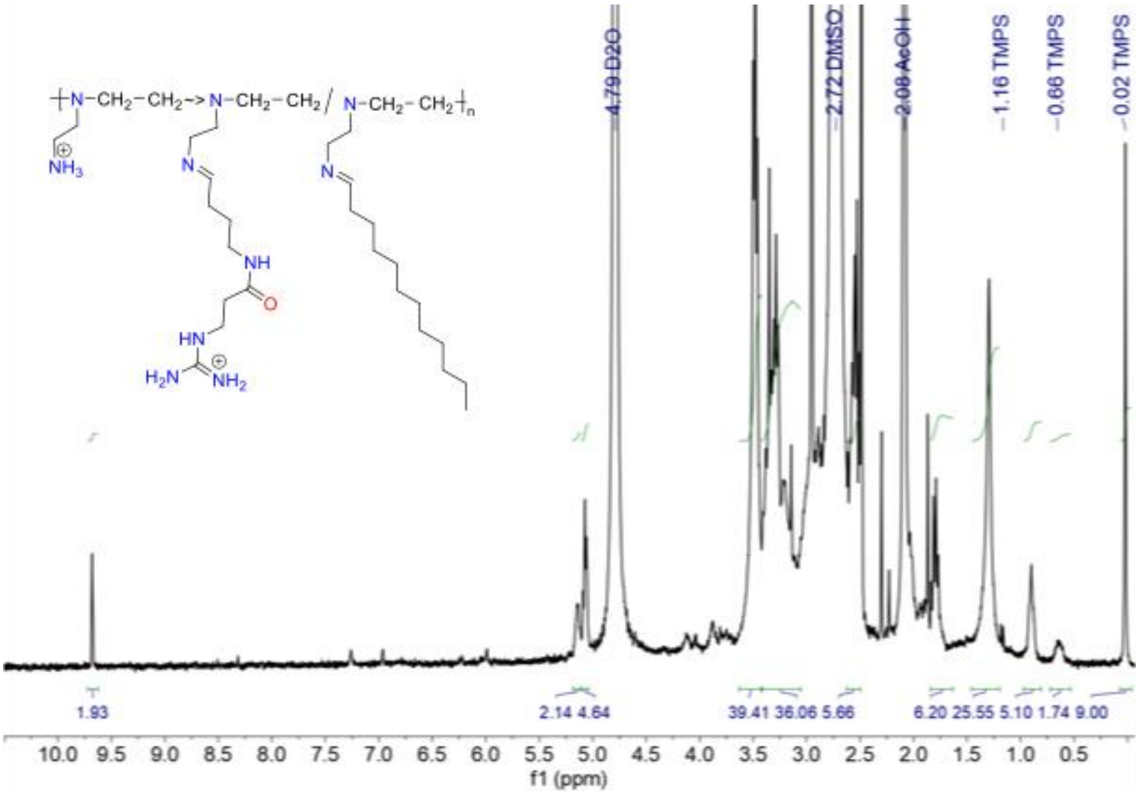

***Figure S14.** **^{1}H NMR spectra of p[PEI-mod-T1$_{0.34}$T3B$_{0.6}$] (PEIT1T3B)** in acetate buffer (100 mM, pH 3.0) in D_2O. Sodium 3-(trimethylsilyl)propane-1-sulfonate (**TMPS**) used as an internal reference.*

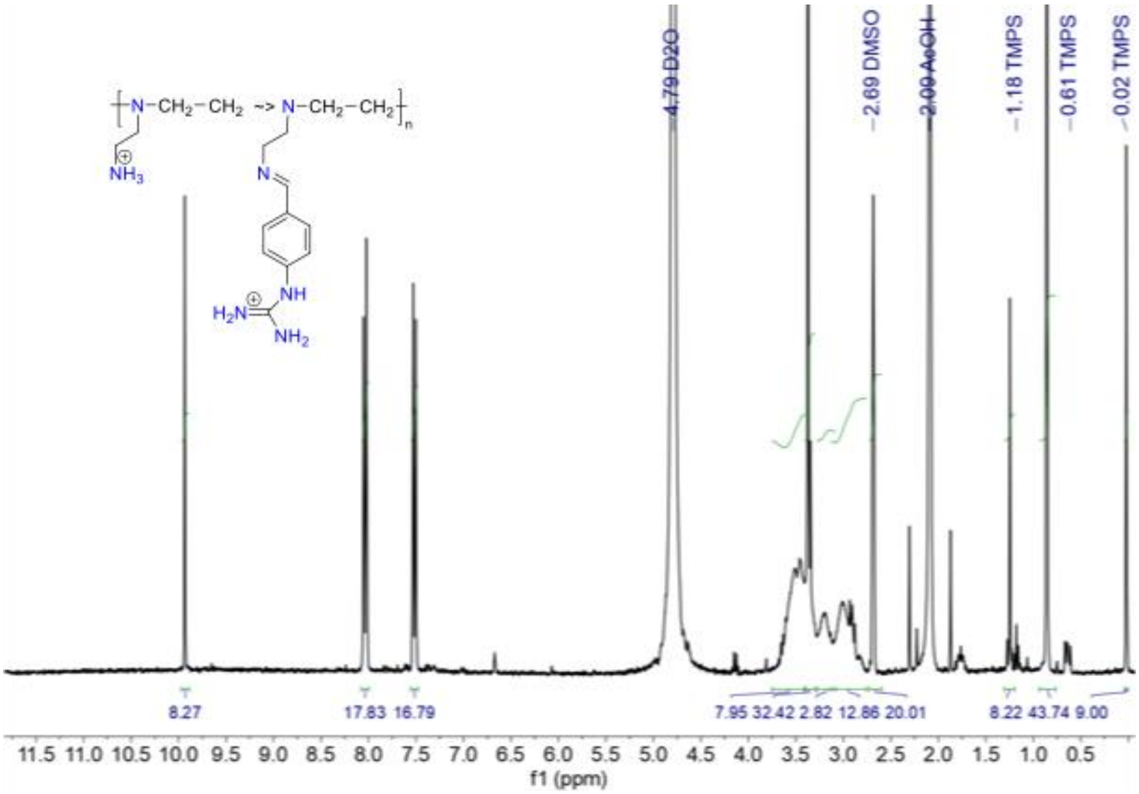

Figure S15. ^{1}H NMR spectra of p[PEI-mod-T2$_{0.19}$] (PEIT2) *in acetate buffer (100 mM, pH 3.0) in D_2O. Sodium 3-(trimethylsilyl)propane-1-sulfonate (**TMPS**) used as an internal reference.*

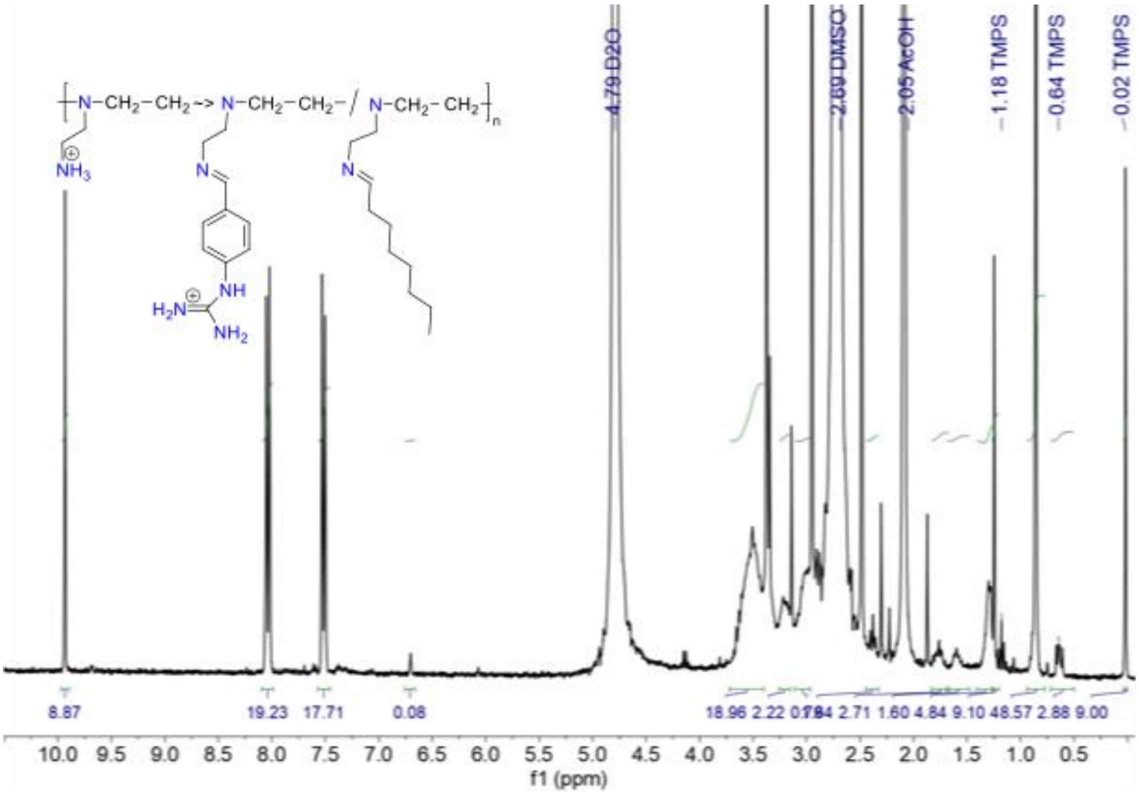

Figure S16. ^{1}H NMR spectra of p[PEI-mod-T2$_{0.15}$T3A$_{0.60}$] (PEIT2T3A) *in acetate buffer (100 mM, pH 3.0) in D_2O. Sodium 3-(trimethylsilyl)propane-1-sulfonate (**TMPS**) used as an internal reference.*

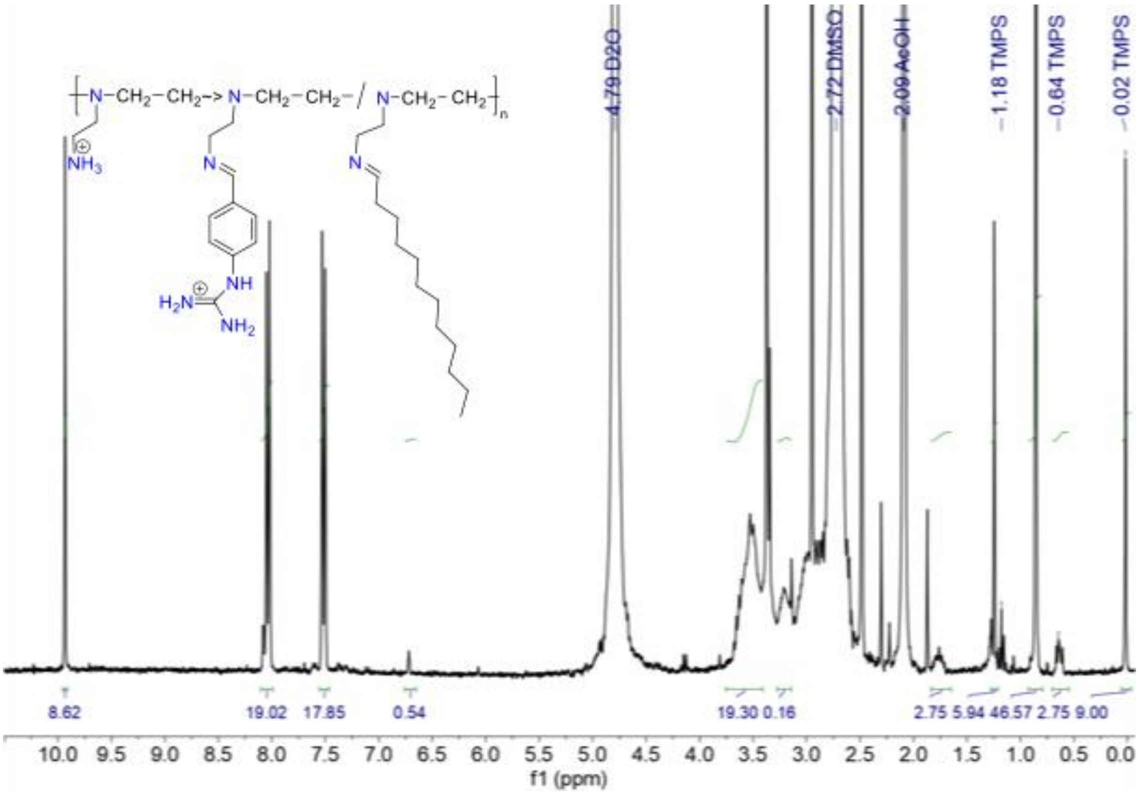

Figure S17. ¹H NMR spectra of p[PEI-mod-T2$_{0.17}$T3B$_{0.60}$] (PEIT2T3B) *in acetate buffer (100 mM, pH 3.0) in D_2O. Sodium 3-(trimethylsilyl)propane-1-sulfonate (**TMPS**) used as an internal reference.*

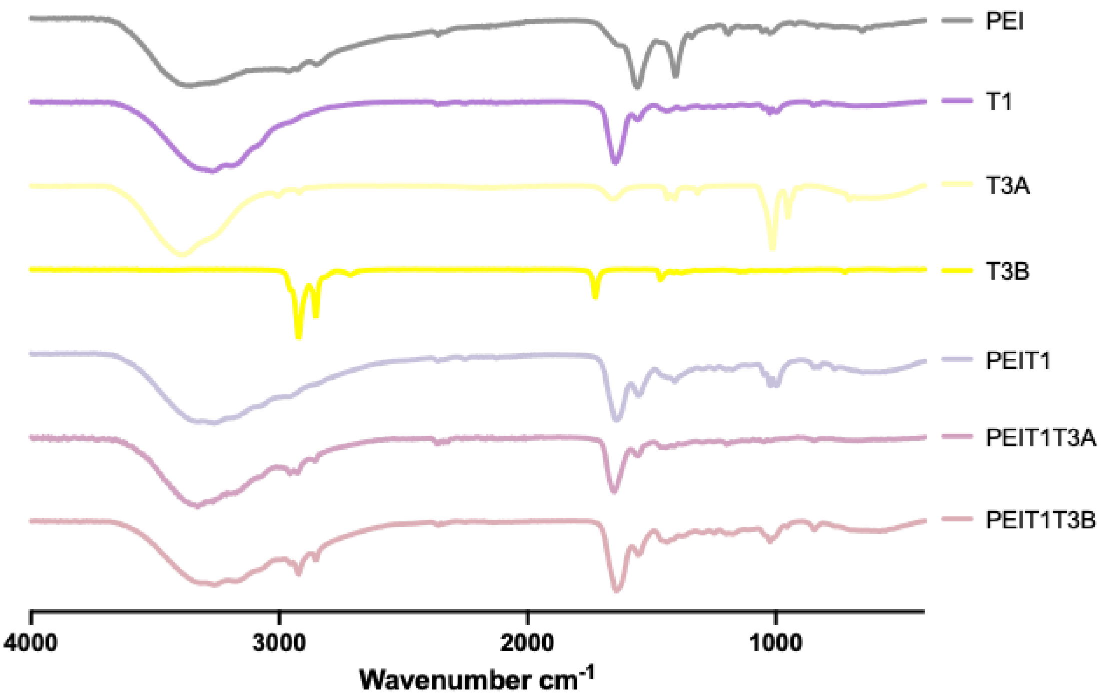

Figure S18. IR spectra of PEI and its functionalization with T1 or T1/T3 mixtures*. T1, T3A and T3B shown for comparison.*

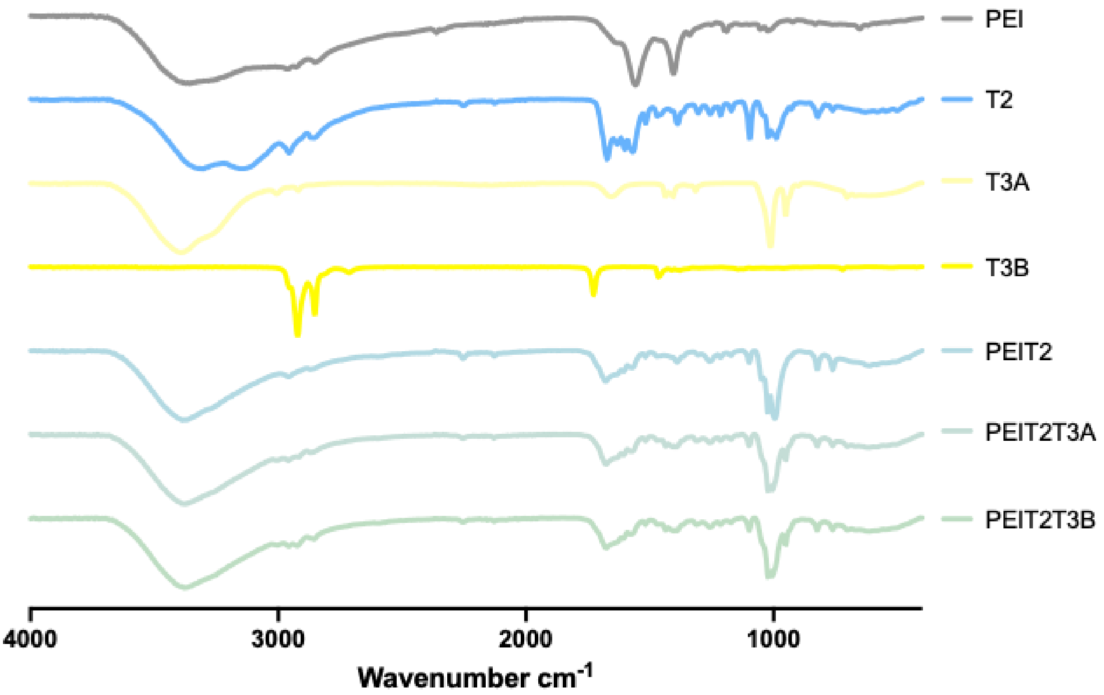

Figure S19. IR spectra of PEI and its functionalization with T2 or T2/T3 mixtures. *T2, T3A and T3B shown for comparison.*
