# Supplementary material for: Novel PEI-aldehyde conjugates for gene delivery: Promoting chondrogenic differentiation in human mesenchymal stem cells
Source: Mol Ther Nucleic Acids. 2025 Apr 29;36(2):102551. doi: 10.1016/j.omtn.2025.102551 (PMC12141052; doi:10.1016/j.omtn.2025.102551)
Supplement: Document S2. Article plus supplemental information [file mmc2.pdf]

Molecular Therapy
Nucleic Acids
Original Article

Novel PEI-aldehyde conjugates for gene delivery: Promoting chondrogenic differentiation in human mesenchymal stem cells

Diego Miranda-Balbuena,[1] Alba Ramil-Bouzas,[1] Naiara Doldán-Mata,[1] Junquera López-Seijas,[1,2] Juan Fafián-Labora,[1,3] Ibán Lamas-Criado,[2] Jose-Ramón Caeiro-Rey,[4] Paco Fernández-Trillo,[1,5] and Ana Rey-Rico[1,2]

[1]Centro Interdisciplinar de Química e Bioloxía - CICA, Universidade da Coruña, 15071 A Coruña, Spain; [2]Departamento de Biología, Facultade de Ciencias, Universidade da Coruña, 15071 A Coruña, Spain; [3]Departamento de Fisioterapia, Medicina y Ciencias Biomédicas, Facultad de Ciencias de la Salud, Universidade da Coruña (UDC), Instituto de Investigación Biomédica de A Coruña (INIBIC), Complexo Hospitalario Universitario de A Coruña (CHUAC), Servizo Galego de Saúde (SERGAS), 15006 A Coruña, Spain; [4]Departamento de Cirugía Ortopédica y Traumatología, Complexo Hospitalario Universitario de Santiago de Compostela (CHUS), Universidade de Santiago de Compostela (USC), 15706 Santiago de Compostela, Spain; [5]Departamento de Química, Facultade de Ciencias, Universidade da Coruña, 15071 A Coruña, Spain

Mesenchymal stem cell (MSC) gene therapy holds significant potential for regenerative medicine, especially for treating conditions such as cartilage damage. Still, finding appropriate vectors to achieve a safe and efficient gene delivery remains a challenge. This study explores the development of novel polyethyleneimine (PEI)-based polymers functionalized with both cationic guanidinium and hydrophobic aldehyde groups for efficient transfection to human MSCs (hMSCs). PEI was chemically modified with guanidinium-(3-guanidin-N-(3-oxopropyl)propanamide [T1]) and 1-(4-formylphenyl)guanidine [T2]) and hydrophobic (octanal [T3A] and dodecanal [T3B]) aldehydes. Polyplexes were formed by the complexation of PEI-aldehyde conjugates with plasmids encoding for β-galactosidase (p*lacZ*), green fluorescent protein (pGFP), and the chondrogenic transcription factor SOX9 (p*sox9*), and demonstrated efficient DNA complexation and protection. Among the formulations, PEI functionalized with the cationic (T2) and hydrophobic (T3A) aldehydes (PEIT2T3A) exhibited a superior transfection efficiency and biocompatibility, significantly enhancing the expression of target genes in hMSCs. Importantly, PEIT2T3A/p*sox9* polyplexes successfully promoted the chondrogenic differentiation of hMSCs, as evidenced by the increased expression of chondrogenic markers (SOX9, type-II collagen [COLII], and aggrecan [ACAN]) and proteoglycan deposition in aggregate cultures, while mitigating the low cell viability found with unmodified PEI. These findings suggest that PEIT2T3A is a promising non-viral vector for targeted gene delivery and hMSC-based regenerative medicine applications.

INTRODUCTION

Gene therapy and regenerative medicine hold immense potential for treating a wide range of diseases and injuries by enabling precise genetic modifications of target cell populations.[1] By directly altering the genetic material within a cell, these therapies can correct genetic defects, promote the regeneration of damaged tissues, and modulate cellular functions in a targeted manner.[2] Of note, a critical aspect of these therapies is the development of efficient and safe gene delivery systems, which are essential for transferring therapeutic genes into the cells of interest.[3]

Among the various gene delivery systems, polyethyleneimine (PEI) has emerged as one of the most promising non-viral vectors for gene therapy.[4,5] PEI's popularity relies on its strong DNA-binding capability, which helps form compact polyplexes with plasmid DNA. Additionally, its proton-sponge effect promotes endosomal escape, a key step in ensuring that the DNA reaches the cell nucleus without being degraded.[4,5] However, despite the effectiveness of PEI as a DNA nanocarrier, its high cytotoxicity and relatively low transfection efficiency in certain cell types significantly precludes its widespread use in clinical applications.[5] As a matter of fact, some studies have described <10% transfection efficiency in human mesenchymal stem cells (hMSCs) with PEI compared with >90% in HEK293 cells.[6] These limitations have stressed the need to find new alternatives to enhance PEI performance while mitigating its cytotoxic effects.

To address these challenges, recent advancements in polymer chemistry have opened new avenues for the design of PEI-based gene

Received 9 January 2025; accepted 25 April 2025;
https://doi.org/10.1016/j.omtn.2025.102551.

Correspondence: Paco Fernández-Trillo, Gene and Cell Therapy Research Group (G-CEL), Centro Interdisciplinar de Química e Bioloxía - CICA, Universidade da Coruña, 15071 A Coruña, Spain.
E-mail: f.ftrillo@udc.es
Correspondence: Ana Rey-Rico, Gene and Cell Therapy Research Group (G-CEL), Centro Interdisciplinar de Química e Bioloxía - CICA, Universidade da Coruña, 15071 A Coruña, Spain.
E-mail: ana.rey.rico@udc.es

Published by Elsevier Inc. on behalf of The American Society of Gene and Cell Therapy.
This is an open access article under the CC BY license (http://creativecommons.org/licenses/by/4.0/).

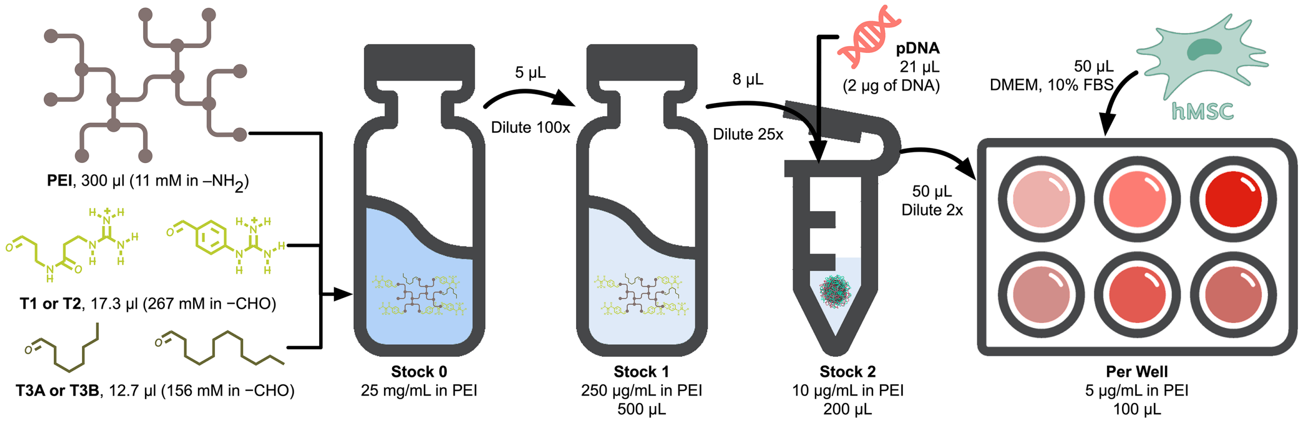

Figure 1. Schematic representation of the formation and employment of PEI-based polyplexes for the transfection of hMSCs
Cationic/hydrophobic aldehyde ratio of 70/30, N/P ratio of 7, and [PEI] of 5 μg/mL.

delivery systems. One strategy involves the functionalization of PEI with cationic groups,[7] such as guanidinium, which is known for its strong ionic interactions with nucleic acids. Guanidinium groups mimic the arginine-rich domains of naturally occurring DNA-binding peptides and proteins, thereby enhancing the ability of PEI to bind DNA and penetrate cell membranes.[7–9] Additionally, hydrophobic modifications have been introduced to PEI to make it an amphiphilic molecule, improving its interaction with cell membranes and, therefore, facilitating endosomal escape by destabilizing and fusing with the endosomal membrane.[10] Introducing hydrophobic moieties also reduces cytotoxicity by neutralizing polycation surface charge and decreasing its lytic activity.[4,11] In particular, the incorporation of hydrophobic aldehydes, such as octanal (T3A) and dodecanal (T3B) into PEI molecules has been shown to alter the polymer's hydrophilic-hydrophobic balance, thereby enhancing its cellular uptake and transfection efficiency.[12,13]

Although the functionalization of PEI with either cationic or hydrophobic groups has been explored before,[10,11,14] the use of these specific aldehydes and, in particular, their combination is yet to be investigated. Furthermore, while some PEI modifications have led to improved transfection efficiency in various cell lines, their effectiveness in more clinically relevant cell types, such as hMSCs, has not been fully explored.[6,14] As hMSCs are of particular interest in regenerative medicine applications due to their multipotent differentiation capacity and feasibility to induce tissue remodeling,[15,16] efficient gene delivery to this population could significantly enhance their therapeutic potential.[15,17]

In this study, we aim to increase PEI's gene delivery ability by synthesizing and characterizing a series of PEI derivatives functionalized with guanidinium aldehydes—3-guanidin-N-(3-oxopropyl)propanamide (T1) and 1-(4-formylphenyl)guanidine (T2)—and hydrophobic aldehydes—T3A and T3B.[18,19] These modifications were selected based on their potential to enhance DNA binding, cellular uptake, and transfection efficiency while reducing the cytotoxicity commonly associated with unmodified PEI.[18,20] We systematically evaluated the ability of these modified PEIs to form stable polyplexes with a reporter DNA plasmid, protecting the genetic cargo from enzymatic degradation, and efficiently transfecting hMSCs (Figure 1). Additionally, we investigated the potential of these modified PEIs to facilitate the chondrogenic differentiation of hMSCs by delivering a plasmid encoding the chondrogenic transcription factor SOX9, a key regulator of cartilage formation.[21] Our findings demonstrate that these functionalized PEI polymers, combining cationic and hydrophobic properties, significantly improve gene delivery and promote hMSC differentiation.

RESULTS

Physicochemical characterization and complexation capacity of PEI

First, the size and zeta potential of PEI and PEI polyplexes with p*lacZ*, a reporter plasmid encoding for β-galactosidase, were characterized (Figure 2A). The size of PEI complexes with p*lacZ* (N/P (nitrogen/phosphate ratio) 14, 2.5 μg/mL of DNA) (348.33 ± 4.76 nm) was significantly lower than that of PEI alone (421.18 ± 3.41 nm) ($p \leq 0.0042$). A similar trend was observed for the zeta potential, the charge of the polyplex being significantly lower (34.91 ± 2.1 mV) than that obtained with unmodified PEI (43.64 ± 5.32 mV) ($p \leq 0.0427$).

The ability of PEI to efficiently condense p*lacZ* was then evaluated using the SYBR green dye exclusion assay (Figure 2B). Increasing the concentration of PEI led to a higher complexation of the plasmid, as observed by a decrease in the fluorescence intensity. All the fluorescence values for the polyplexes were significantly different than those observed for free DNA (2.5 μg/mL, $p < 0.0001$). The fluorescence intensity relative to the free plasmid DNA ranged from 46.15% at the lowest concentration of PEI (1 μg/mL) to 13.81% at its highest concentration (50 μg/mL), with statistically significant differences between these two concentrations ($p < 0.0001$). No statistical differences in complexation were observed by increasing the

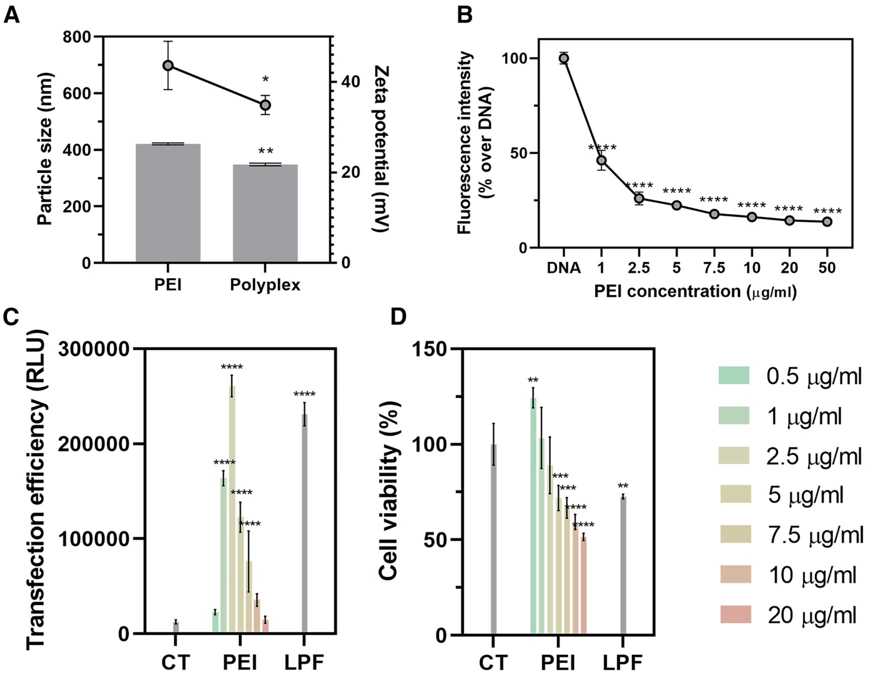

Figure 2. Unmodified PEI characterization

(A) Particle size (bars) and zeta potential (dots) of PEI and PEI-placZ polyplex. N/P 14: [PEI] = 5 μg/mL and [placZ] = 2.5 μg/mL. (B) DNA p*lacZ* complexation efficiency of PEI polyplexes formed at different concentrations. (C) β-Galactosidase activity and (D) cell viability after transfection with PEI-placZ at various concentrations in iMSCs. The commercial reagent Lipofectamine (LPF) was used as positive transfection control. [p*lacZ*] = 2.5 μg/mL. **p* < 0.05, ***p* < 0.01, ****p* < 0.001, and *****p* < 0.0001, when compared with respective controls. Data are expressed as mean of quadruplicates, and error bars indicate standard deviation.

concentration above 7.5 μg/mL of PEI (17.85% fluorescence intensity) ($p \geq 0.57$).

Optimization of PEI concentration for *in vitro* transfection of iMSCs

In vitro transfection of immortalized mesenchymal stem cells (iMSCs) with PEI showed comparable levels of β-galactosidase activity to those achieved with the commercial reagent Lipofectamine (LPF) (231,107.75 ± 12,108.02 RLU [relative luminiscence units]) (Figure 2C). These values were significantly higher than those recorded for the negative control (i.e., untreated cells, CT) when using concentrations of PEI from 1 μg/mL to 7.5 μg/mL. This increase in transfection was particularly relevant at 2.5 μg/mL of PEI ($p < 0.0001$). When testing cell viability after transfection, no differences were observed between the negative control and PEI at concentrations below 5 μg/mL (71.89 ± 6.57%) ($p \geq 0.39$). In contrast, a reduction in cell viability was evidenced upon transfection with PEI at concentrations above 5 μg/mL or when the commercial reagent LPF was used (72.62 ± 1.13%) ($p \leq 0.012$) (Figure 2D). In all cases, DNA concentration was 2.5 μg/mL.

In vitro transfection of hMSCs with PEI-based polymer formulations

Initial transfection of placZ polyplexes with PEI-based polymers (PEIT1, PEIT1T3A, PEIT1T3B, PEIT2, PEIT2T3A, PEIT2T3B, PEIT3A, and PEIT3B) was performed at 1, 5, and 10 μg/mL of PEI. The cationic/hydrophobic aldehyde ratio was maintained at 70/30 while the DNA concentration was 2.5 μg/mL. hMSC primary cultures isolated from bone marrow aspirates were used in this case and showed the specific profiles of expression characteristic from hMSCs (Figure S1), being positive for the three MSC-positive surface markers (CD90, CD73, and CD105). The expression of CD90 was >96%, while CD105 and CD73 showed lower response (~30%). Conversely, the expression of hematopoietic surface markers (CD34 and CD45) showed values <1% (Figure S1). This experiment indicated that the highest transfection efficiencies were observed between 5 and 10 μg/mL (Figure S2), as opposed to what we observed for unmodified PEI, showing the highest transfection efficiencies between 1 and 5 μg/mL (Figure 2C).

Thus, this experiment was repeated at 5, 7.5, and 10 μg/mL (Figures 3A and 3B). Gene transfer efficiency of hMSCs monolayers treated with p*lacZ* polyplexes always showed a superior efficiency when using 5 μg/mL of PEI rather than at any other concentration tested ($p \leq 0.026$). A similar trend was observed when measuring cell viability, with the highest cell viability values achieved at 5 μg/mL of PEI ($p \leq 0.001$). Of note, PEIT2T3A always led to the highest transfection values among all formulations studied (up to 8.2-fold increase regarding unmodified PEI).

Next, a similar experiment was conducted, fixing the concentration of PEI at 5 μg/mL and the N/P ratio at 14 (2.5 μg/mL of DNA) while varying this time the cationic/hydrophobic aldehydes ratio (70/30, 87.5/12.5, and 93/7) for the PEIT2, PEIT2T3A, PEIT2T3B, PEIT3A, and PEIT3B formulations (Figures 3C and 3D). Polyplexes formed at a 70/30 ratio yielded the highest transfection efficiency ($p \leq 0.046$ and $p \leq 0.04$ compared with 87.5/12.5 and 93/7 ratios, respectively). Irrespective of the cationic/hydrophobic aldehyde ratio tested, PEIT2T3A always led to the highest transfection values (up to 7.7-fold increase in β-galactosidase activity). Cell viability percentages were around 85% for 70/30 and 87.5/12.5 ratios, exhibiting a slight decrease at the highest ratio (93/7) tested (~75%; $p \leq 0.03$ compared with 70/30 ratio). To ensure that the observed transfection efficiencies were not the result of any free aldehydes, solvents, or DNA that may be present in the solution, a similar experiment was performed with each of these components. No significant

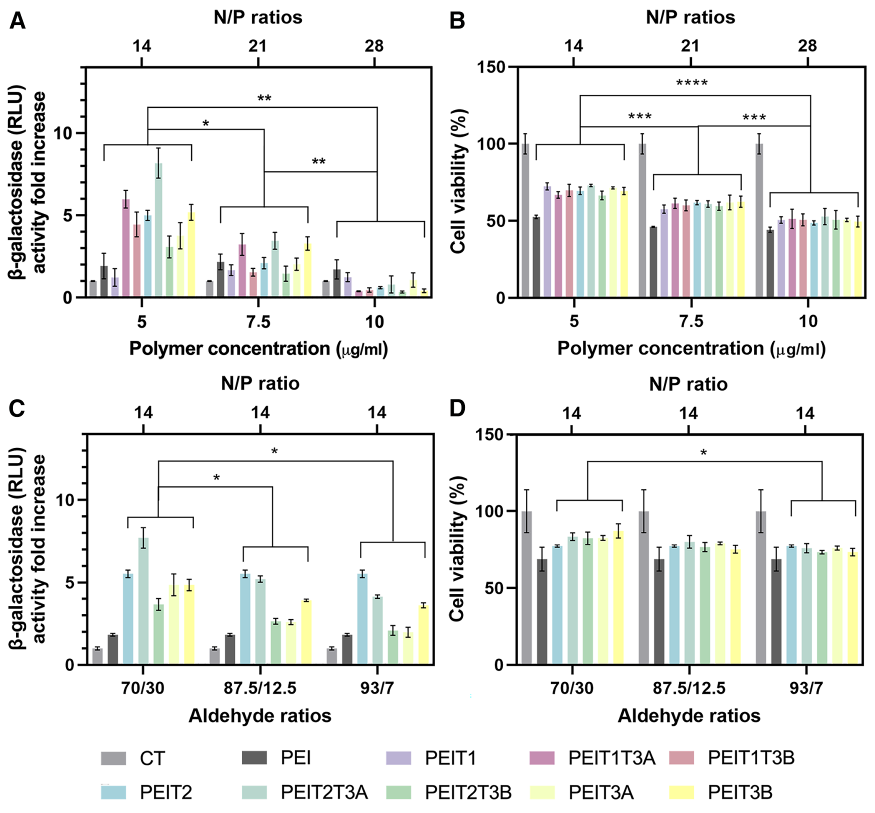

Figure 3. Effect of transfectant concentration and aldehyde ratio on transfection efficiency and cell viability

(A) β-galactosidase activity and (B) cell viability after transfection with different polyplexes (PEI, PEIT1, PEIT1T3A, PEIT1T3B, PEIT2, PEIT2T3A, PEIT2T3B, PEIT3A, and PEIT3B) (p*lacZ*) at various concentrations (5 μg/mL, 7.5 μg/mL, and 10 μg/mL) and N/P ratios (14, 21, and 28) in hMSCs. Cationic/hydrophobic aldehyde ratio = 70/30. (C) β-Galactosidase activity and (D) cell viability after transfection with different polyplexes (PEI, PEIT2, PEIT2T3A, PEIT2T3B, PEIT3A, and PEIT3B) at various cationic/hydrophobic aldehyde ratios (70/30, 87.5/12.5, and 93/7) and N/P ratio of 14 in hMSCs. [PEI] = 5 μg/mL and [p*lacZ*] = 2.5 μg/mL. *$p < 0.05$, **$p < 0.01$, ***$p < 0.001$, and ****$p < 0.0001$, when compared denoted groups. Data are expressed as mean of quadruplicates, and error bars indicate standard deviation.

changes in transfection efficiency, or an overall decrease below 95% of viability, were observed for the aldehydes, solvents, or DNA when compared to the untreated cells (Figure S3).

Finally, to elucidate the best N/P ratio of polyplexes for the PEIT2, PEIT2T3A, PEIT2T3B, PEIT3A, and PEIT3B formulations, a final transfection with p*lacZ* was carried out (Figures 4A and 4B) by maintaining the PEI concentration at 5 μg/mL and the cationic/hydrophobic aldehyde ratio at 70/30 while modifying the DNA concentration (5, 2.5, and 1.25 μg/mL of DNA, respectively) to obtain N/P ratios of 7, 14, and 21. Polyplexes formed at an N/P ratio of 7 yielded the highest levels of transfection efficiency ($p \leq 0.05$ compared with 14 and 21 N/P ratios). Again, PEIT2T3A led to the highest levels of transfection (up to 12.2-fold increase in β-galactosidase activity).

Finally, a similar transfection experiment was conducted in hMSC monolayer cultures but using a different reporter plasmid p*GFP* that encodes for expression of a green fluorescent protein. Transfection efficiency was evaluated by flow cytometry (Figures 4C and 4D). Similar to what we observed with p*lacZ*, *pGFP* polyplexes formed at an N/P ratio of 7 led to higher transfection values ($p \leq 0.05$ compared with 14 and 21 N/P ratios), PEIT2T3A again reaching the highest values (up to 26-fold increase in GFP expression). An analysis of cell viability after transfection showed the highest percent of cell survival at this lowest N/P ratio tested ($p \leq 0.015$ compared with 14 and 21) (Figures 4B and 4D).

Chondrogenic differentiation of hMSCs upon transfection with polyplexes

hMSC aggregates were transfected with selected polymer formulations (PEIT2, PEIT2T3A, PEIT3A, and PEIT3B) and a plasmid encoding for the chondrogenic transcription factor SOX9[22] (p*sox9*) and cultured in chondrogenic medium (CT) for 21 days. Control conditions included untreated aggregates cultured in CT or cultured in the same medium but transfected with p*sox9* using unmodified PEI. Transgene SOX9 expression was observed for all hMSC aggregates, as a result of the effective chondrogenic differentiation of the cells upon continuous induction particularly when p*sox9* was provided to the cells (Figures 5 and S4). Expression of this chondrogenic factor reached its maximum when transfected with PEIT2T3A (162.74 ± 1.71 mean intensity), exhibiting drastic differences when compared with untreated aggregates (Figure 5A, CT) or those cells transfected with unmodified PEI ($p < 0.0001$). These findings were further corroborated by an analysis of *sox9* profiles by real-time RT-PCR, where PEIT2T3A led to the highest levels of expression (up to 5.9 -fold increase; $p \leq 0.0002$ compared with CT or PEI-transfected cells) (Figure 6A).

Chondrogenic differentiation was observed in all the samples as indicated by the intense toluidine blue staining (TB, matrix proteoglycans) and type-II collagen (COLII) deposition (Figures 5 and S4), especially in those aggregates transfected with PEIT2T3A (166.66 ± 1.95 mean intensity for COLII and 100.92 ± 6.89 mean intensity for TB), showing again statistically significant differences when compared with untreated controls (CT) or PEI transfected aggregates ($p < 0.0001$) (Figures 5 and S4). Real-time RT-PCR analyses also showed an up-regulation of COLII and ACAN (gene related to aggrecan production, the most abundant proteoglycan in cartilage), especially upon transfection with PEIT2T3A (up to 5.2-fold increase

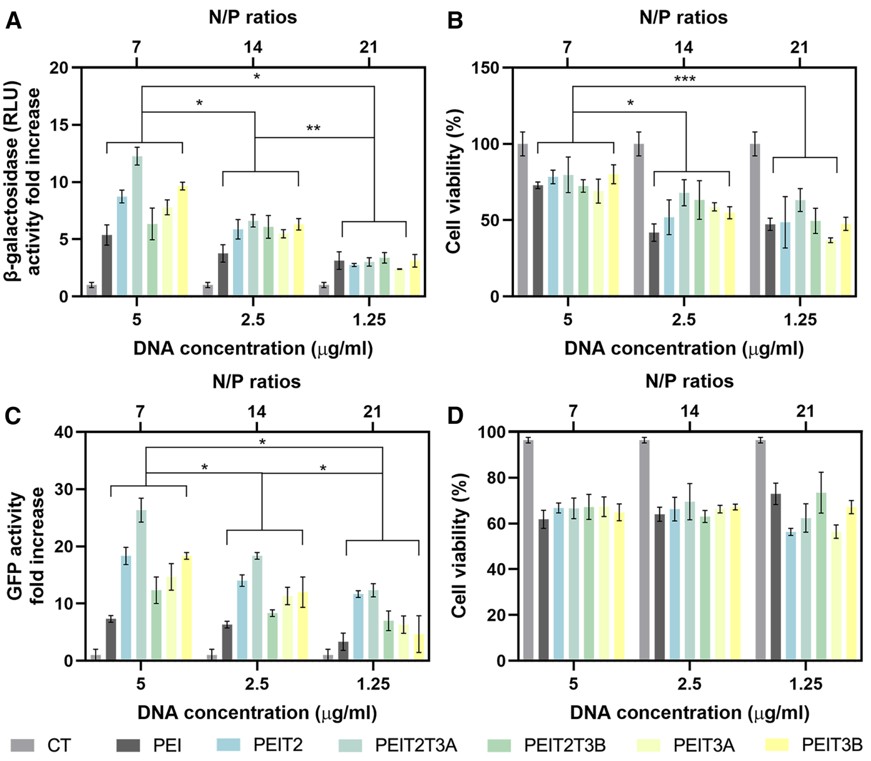

Figure 4. Effect of DNA concentration and N/P ratio on transfection efficiency and cell viability

(A) β-galactosidase activity and (B) cell viability after transfection with different polyplexes (PEI, PEIT1, PEIT1T3A, PEIT1T3B, PEIT2, PEIT2T3A, PEIT2T3B, PEIT3A, and PEIT3B) (placZ) at various DNA concentrations (5 μg/mL, 2.5 μg/mL, and 1.25 μg/mL) and N/P ratios (7, 14, and 21) in hMSCs (C) GFP expression fold increase and (D) cell viability after transfection with different polyplexes (PEI, PEIT1, PEIT1T3A, PEIT1T3B, PEIT2, PEIT2T3A, PEIT2T3B, PEIT3A, and PEIT3B) (pGFP) at various DNA concentrations (5 μg/mL, 2.5 μg/mL, and 1.25 μg/mL) and N/P ratios (7, 14, and 21) in hMSCs. Cationic/hydrophobic aldehyde ratio = 70/30 and [PEI] = 5 μg/mL. *$p < 0.05$, **$p < 0.01$, and ***$p < 0.001$, when compared denoted groups. Data are expressed as mean of quadruplicates, and error bars indicate standard deviation.

for COLII and ACAN; $p < 0.0056$ compared with CT or PEI transfected cells) (Figure 6A).

Genetic modification of aggregates via PEIT2T3A led to a reduction in COLI immunoreactivity (52.95 ± 2.83 mean intensity) when compared with those pellets transfected with PEI (75.7 ± 3.29 mean intensity; ($p < 0.0001$) or with untreated aggregates (CT, 78.99 ± 4.17 mean intensity; $p < 0.0001$) (Figures 5 and S4). However, these mean differences in COLI expression were not enough to be considered significant ($p \leq 0.0835$) (Figure 6A). Yet, an increase of COLX immunoreactivity was also evidenced in cell aggregates transfected with PEIT2T3A (110.68 ± 3.15 mean intensity) when compared to the untreated cells (CT, 85.46 ± 3.23 mean intensity, $p < 0.0001$). However, this difference was more notable with those aggregates transfected with PEI (123.87 ± 3.21 mean intensity, $p < 0.0001$) (Figures 5 and S4). Still, these differences were not detected at the mRNA level ($p \leq 0.9738$) (Figure 6A). Lastly, an analysis of cellularity in cell aggregates by H&E staining evidenced lower cell densities in those pellets transfected with PEIT2T3A (7.5 ± 1.34 cells/mm^2) compared with CT (9.58 ± 1.28 cells/mm^2) or PEI ((9.19 ± 1.22 cells/mm^2), even though statistically significant differences were not observed ($p \geq 0.39$) (Figures 5 and S4).

These data were further corroborated by analyzing the proteoglycan contents in the aggregate cultures standardized to the total protein (Figure 6B) or to the DNA contents (Figure 6C). SOX9 overexpression in hMSC aggregates via PEIT2T3A promoted an increase in proteoglycan contents (up to a 2.1-fold difference compared with CT; $p < 0.0001$ and up to 1.4-fold difference compared with PEI; $p < 0.0024$) (Figure 6B). A similar trend was observed when normalizing the number of proteoglycans to the DNA contents (Figure 6C), leading those pellets transfected with PEIT2T3A to a 2.2-fold difference compared with CT ($p < 0.0001$) and to a 1.5-fold difference compared with PEI ($p < 0.0001$).

Cell internalization of PEIT3T2E

To evaluate the endocytosis pathways of polyplexes in hMSCs, we quantified the transgene expression (*lacZ*) upon transfection with PEIT2T3A after treatment with different endocytosis inhibitors (Figure 7A). A significant reduction in β-galactosidase activity was evidenced in those cells pre-treated with genistein and methyl-β-cyclodextrin (4.4 and 4.6 times less, respectively) when compared with cells non-treated with inhibitor ($p < 0.001$ in both cases). Confocal microscopy with Cy3-labeled p*lacZ* PEIT2T3A polyplexes in hMSC cultures (Figure 7B) indicated a higher co-localization of this plasmid with AlexaFluor488-labeled cholera toxin than with AlexaFluor488-labeled transferrin (Figure 7B).

Physicochemical characterization and complexation capacity of PEI and PEIT2T3A

Finally, the size, zeta potential of PEI and PEIT2T3A polymers and polyplexes, and their complexation and protection capacity was evaluated (Figure S5). Both PEIT2T3A polymers (440.06 ± 3.71) and polyplexes (364.84 ± 3.32) were significantly bigger than their PEI counterparts ($p \leq 0.0014$ and $p \leq 0.0034$, respectively). However, no significant differences were observed in terms of zeta potential when these systems were compared with unmodified PEI ($p \geq 0.74$). As before, the ability of both polymers to efficiently condense p*lacZ* was evaluated using the SYBR green dye exclusion assay (Figure S5B). A significant reduction in the percentage of free

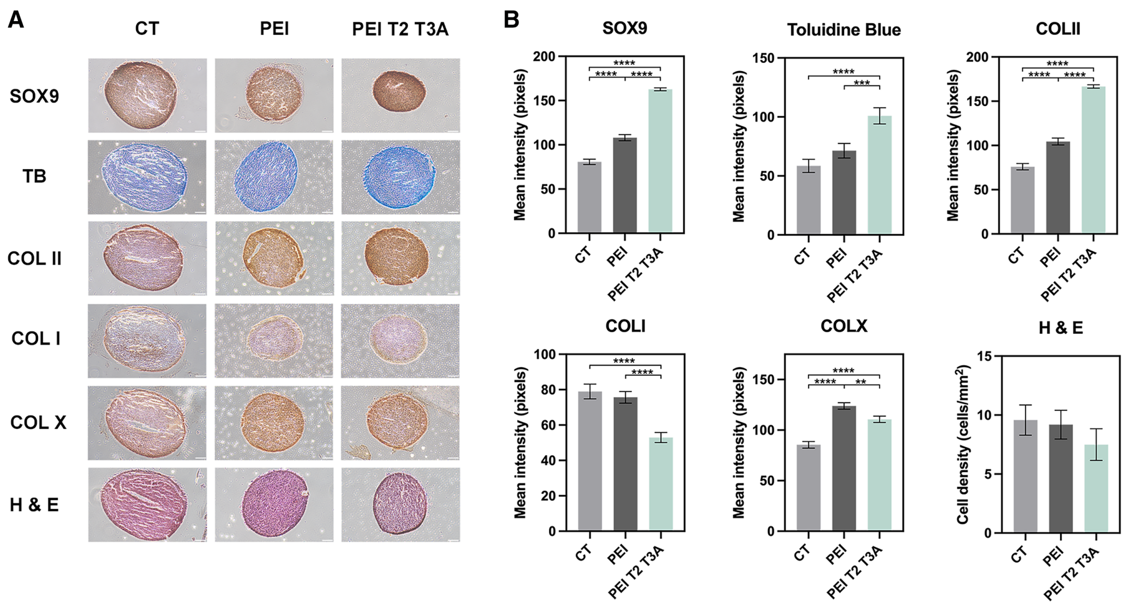

Figure 5. Immunohistochemical and histological analyses of chondrogenesis

Immunohistochemical and histological analyses of hMSC aggregates cultured in chondrogenic medium (CT; negative control) and transfected with psox9 via PEI or PEIT2T3A. Samples were kept in culture for 21 days and processed for (A) immunodetection of SOX9, type-I collagen (COLI), type-II collagen (COLII), type-X collagen (COLX), toluidine blue (TB), and H&E (all representative images; magnification 10×; scale bars: 100 μm). (B) Histomorphometric analyses of the previously mentioned conditions. Cationic/hydrophobic aldehyde ratio = 70/30, [PEI] = 5 μg/mL and [placZ] = 5 μg/mL. **$p < 0.01$, ***$p < 0.001$, and ****$p < 0.0001$, when compared with denoted groups. Data are expressed as mean of triplicates, and error bars indicate standard deviation.

DNA percentage was observed for PEI and PEIT2T3A at all the N/P ratios studied ($p < 0.0001$ compared with free DNA control). However, no differences between PEI and PEIT2T3A were evidenced with the fluorescence intensity ranging from ∼25% to ∼13% at N/P ratios from 7 to 21 ($p \geq 0.95$) in both cases. The same tendency was observed in the DNase protection assay, with a stronger DNA band as the N/P ratio for PEI and PEIT2T3A polyplexes increased (Figure S5C). As expected, and in a further DNase protection assay, practically no band intensity was observed between N/P ratios of 0.25–2 (Figure S5D).

DISCUSSION

The primary aim of this study was to enhance the transfection efficiency of the gold standard polymer PEI in MSCs through its modification with various cationic and hydrophobic aldehydes. The cationic aldehydes T1 and T2 were selected due to their strong ionic interactions with nucleic acids. The guanidinium groups in these aldehydes are known to mimic the arginine-rich domains of cell-penetrating peptides, enhancing DNA complexation and facilitating cellular membrane penetration.[7,9] In the case of the hydrophobic aldehydes, T3A and T3B were incorporated to introduce hydrophobic domains into the PEI structure. Hydrophobic modifications were expected to improve interactions with cell membranes, promote endosomal escape (by making the whole molecule amphiphilic), and reduce the cytotoxic effects of PEI.[4,11] We postulated that combining these aldehydes would improve the formation of stable polyplexes with DNA plasmids, protecting the genetic cargo from degradation and promoting efficient gene delivery to hMSCs. Our results demonstrated that these PEI modifications significantly influenced the physicochemical properties of the PEI-based polyplexes, impacting their transfection efficiency, cytotoxicity, and ability to promote the chondrogenic differentiation of hMSCs.

In general, the modification of PEI with guanidinium aldehydes (T1 and T2) is expected to increase DNA interaction and with hydrophobic aldehydes (T3A and T3B) is expected to improve cell entry and reduce cytotoxicity. Also, the modification of PEI with guanidinium aldehyde T2 and hydrophobic aldehyde T3A led to the formation of polyplexes with smaller sizes and lower zeta potentials compared to unmodified PEI (Figure S5A). These results are in good agreement with previous studies where functionalization with amino acids or PEI shielding led to a reduction in size, zeta potential, or both.[14,23] Here, the reduction in size and charge was likely attributed to the steric and electrostatic stabilization provided by the aldehyde modifications. This stabilization, evidenced by the lack of disruption of the polyplexes in the presence of SDS during the electrophoresis assay (Figure S5C), could enhance cellular uptake by minimizing aggregation and reducing cytotoxicity, as evidenced by the increased cell viability after transfection with the functionalized formulations when compared to unmodified PEI (Figures 3, 4, 5, and 6).[24,25] The increased transfection efficiency observed in modified PEI formulations, particularly PEIT2T3A, suggests that the balance between cationic charge and hydrophobicity was critical for efficient gene delivery,[3,4] especially when changing from transfection of iMSCs to

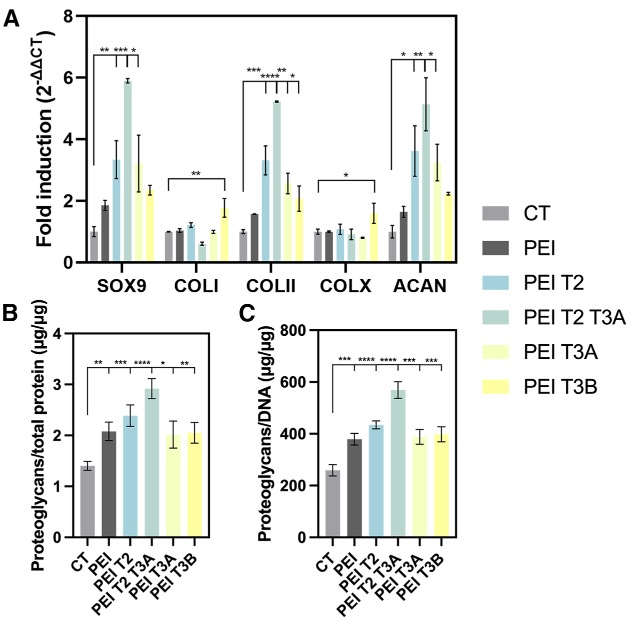

Figure 6. qPCR and biochemistry analyses of chondrogenesis

(A) Real-time RT-PCR analysis of hMSC aggregates cultured in chondrogenic medium and transfected with different polyplexes (PEI, PEIT2, PEIT2T3A, PEIT3A, and PEIT3B) (p*sox9*) after 3 weeks *in vitro*. The genes analyzed included aggrecan (ACAN), the transcription factor SOX9, type-II collagen (COLII), type-X collagen (COLX), and type I collagen (COLI) with GAPDH serving as a housekeeping gene and internal control. Ct values were obtained for each target and GAPDH as a control for normalization, and fold inductions (relative to control aggregates) were measured using the $2^{-\Delta\Delta Ct}$ method. (B) Proteoglycan contents standardized to the protein contents after biochemical analyses of hMSC aggregates cultured in chondrogenic medium and transfected with p*sox9* different polyplexes (PEI, PEIT2, PEIT2T3A, PEIT3A, and PEIT3B) after 3 weeks *in vitro*. (C) Proteoglycan contents standardized to the DNA contents after biochemical analyses of hMSC aggregates cultured in chondrogenic medium and transfected with psox9 different polyplexes (PEI, PEIT2, PEIT2T3A, PEIT3A, and PEIT3B) after 3 weeks *in vitro*. Cationic/hydrophobic aldehyde ratio = 70/30, [PEI] = 5 μg/mL and [placZ] = 5 μg/mL. *$p < 0.05$, **$p < 0.01$, ***$p < 0.001$, and ****$p < 0.0001$. Data are expressed as mean of triplicates, and error bars indicate standard deviation.

primary hMSCs, where higher concentrations (5 μg/mL) were needed due to the lower transfection efficiencies achieved with primary cell cultures.[26,27] Of note, PEIT2T3A formulation, with a 70/30 cationic/hydrophobic aldehyde ratio, displayed the highest transfection efficiency, significantly outperforming unmodified PEI by a factor of 10, followed by PEIT2, PEIT3B, and PEIT3A formulations. The highest efficiency achieved with this formulation compared to other formulations evaluated may be due to the increase in charge without significantly increasing hydrophobicity. In agreement with previous reports[28,29] both unmodified PEI and PEIT2T3A showed DNA protection capacity which increased with higher N/P ratios.

Cytotoxicity of PEI is a well-documented limitation for its application for gene delivery.[30–32] In this study, the modified PEI formulations demonstrated a significant improvement in cell viability, up to 1.4 times, compared to unmodified PEI. This improvement is likely attributed to the reduced cationic charge density and the introduction of hydrophobic moieties, which may mitigate the membrane disruption typically caused by highly cationic polymers.[30] The lower cytotoxicity of PEIT2T3A, combined with its superior transfection efficiency, highlights the potential of this formulation as a safer and more effective gene delivery vector. Notably, these observations were also evidenced when testing in primary cultures of hMSCs, which present more stringent growth requirements and lower proliferative capacity than established cell lines, making them more resistant to transfection and more susceptible to cytotoxic effects.[6]

The ability of aldehyde-modified PEI polyplexes, such as PEIT2T3A, to promote MSCs chondrogenic differentiation is particularly promising when used in cartilage reparative approaches. As a matter of fact, previous studies demonstrated that despite the efficiency of PEI for transfection, it tends to promote a rounded MSC morphology, which can lead to adipogenic differentiation rather than osteogenic or chondrogenic.[33] When used to deliver the chondrogenic factor transforming growth factor β3 (TGF-β3) in bone marrow-derived MSCs, PEI failed to induce robust chondrogenesis of MSCs, as noted by the absence of a significant COLII deposition.[33] In contrast, our formulations, in particular PEIT2T3A, appear to mitigate some of these drawbacks when involved in delivering a plasmid encoding for the key regulator of chondrogenesis SOX9[22] in a 3D hMSCs aggregate culture model.[21] The PEIT2T3A/p*sox9* polyplexes successfully promoted the expression of chondrogenic markers, such as SOX9, COLII, and aggrecan when compared to the basal expression shown by the control or even the expression reached with PEI/p*sox9* polyplexes, while maintaining a moderate expression of fibrocartilage/hypertrophic markers such as type-I and type-X collagen (Figures 5 and 6). Histological and immunohistochemical analyses corroborated these findings, showing enhanced matrix deposition and appropriate chondrogenic differentiation in the treated cell aggregates. These results indicate that the PEIT2T3A formulation enhanced gene delivery and supported the chondrogenic lineage-specific differentiation, making it a promising candidate for cartilage tissue engineering applications.

It is well established that the internalization pathway is a critical step for gene delivery as it determines the efficiency of cellular uptake and the subsequent intracellular trafficking, influencing the overall success of the transfection. The pathway through which polyplexes are internalized can affect their ability to escape endosomal degradation and reach the target cellular compartments, such as the nucleus, for effective gene expression.[28,34] In the present study, PEIT2T3A polyplexes were predominantly internalized via caveolae-mediated endocytosis, with a lesser contribution from clathrin-mediated pathway as inferred from the lower transfection efficiencies values reached after incubation with genistein and methyl-β-cyclodextrin, being both caveolae-related inhibitors (Figure 7). These results are consistent with previous literature about the preferred routes of internalization of cationic polymers[5,35] and with the hydrophobic modification of PEI, which may favor interactions with lipid rafts and caveolae, facilitating cellular entry through this route.[36]

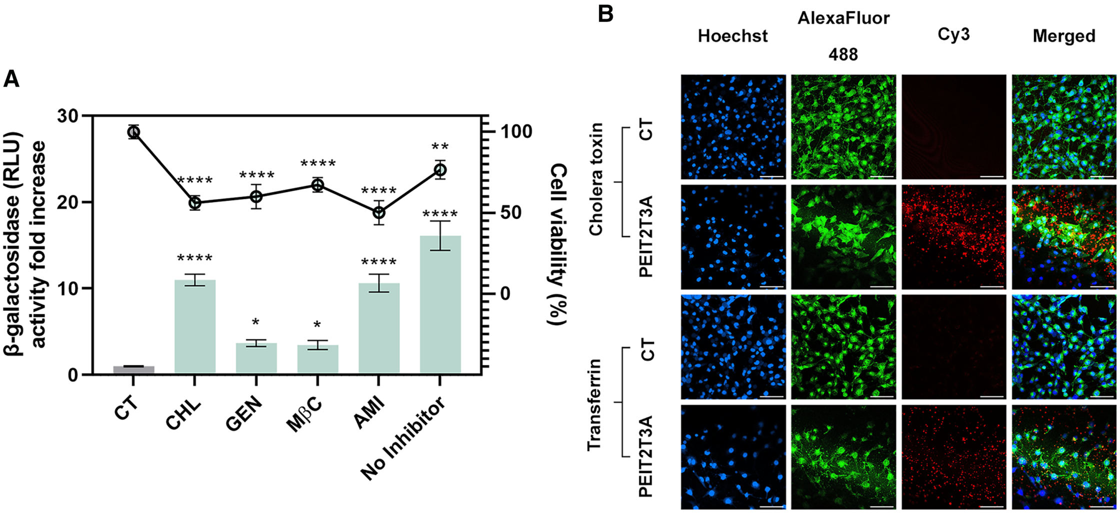

Figure 7. Determination of PEIT2T3A internalization route

(A) β-Galactosidase activity and cell viability of hMSCs pre-treated or not (No Inhibitor) with endocytosis inhibitors: CHL, chlorpromazine; GEN, genistein; MβC, methyl-β-cyclodextrin, and AMI, amiloride) and subsequent transfected with PEI2T3A-placZ polyplexes. (B). Confocal microscopy representative images (scale bars: 100 μm) showing the intracellular distribution of PEIT2T3A-placZ polyplexes in hMSCs. Blue coloring shows cell nuclei stained with Hoechst 33342, red color shows Cy3-labeled-placZ polyplexes, and green coloring shows cells stained with AlexaFluor488-Transferrin or AlexaFluor488-Cholera toxin. Cationic/hydrophobic aldehyde ratio = 70/30, [PEI] = 5 μg/mL and [placZ] = 5 μg/mL. *$p < 0.05$, **$p < 0.01$, and ****$p < 0.0001$. Data are expressed as mean of triplicates, and error bars indicate standard deviation.

MATERIALS AND METHODS

Materials

Unless otherwise stated, all chemicals were obtained from Gibco Thermo Fisher Scientific (Madrid, Spain). Branched PEI (25 kDa) was obtained from Sigma-Aldrich (Madrid, Spain) and modified with two types of cationic guanidinium aldehydes (T1 and T2) synthesized according to protocols described in the literature[20] and two hydrophobic aldehydes (T3A and T3B) from Sigma-Aldrich (St. Louis, MI, USA). Additional reagents included 1,9-dimethyl-methylene blue (DMMB) dye, L-cysteine, toluidine blue, and recombinant TGF-β3 from Sigma-Aldrich (St Louis, MO, USA).

Anti-type X collagen antibody was obtained from Sigma Aldrich (St Louis, MO, USA), and anti-type II collagen (II-II6B3) antibody was purchased from DSHB (Iowa, IA, USA). Anti-SOX9 (E−9) and anti-type I collagen were procured from Santa Cruz Biotechnology (Heidelberg, Germany). Biotinylated secondary antibody (Ig G H + L) along with ABC (Avidin-Biotin Complex) and DAB (3, 3'-diaminobenzidine) reagents were sourced from Vector Laboratories (Alexis Deutschland GmbH, Grünberg, Germany).

Eosin Y and Harris hematoxylin solutions were from Carl Roth (Karlsruhe, Germany), and chondroitin sulfate C was from TRC (Toronto, ON, Canada).

PE-conjugated anti-human CD34, FITC-conjugated anti-human CD45, PE-conjugated anti-human CD73, FITC-conjugated anti-human CD90, FITC-conjugated anti-human CD105, and their respective isotypes were acquired from BD Biosciences (Madrid, Spain).

The WST-1 assay kit and the First Strand cDNA Synthesis Kit for RT-PCR were purchased from Roche (Mannheim, Germany), while the β-Glo assay kit was from Promega (Madison, WI, USA). The Label IT Nucleic Acid Labeling kit Cy3 was obtained from Mirus Bio (Madison, WI, USA). RNeasy Kit was bought from QIAGEN (Hilden, Germany), and PowerUp SYBR Green Master Mix was from Applied Biosystems (Madrid, Spain).

Plasmid pCMV-SPORT-βgal (p*lacZ*; bp 7,853) was obtained from Gibco Thermo Fisher Scientific (Madrid, Spain), plasmid pCMV6-A-GFP (p*GFP*; bp 5,806) was obtained from OriGene Technologies (MD, USA), and pACP-h*sox9* (p*sox9*; bp 6,915) was kindly provided by Prof. M. Cucchiarini.

T1 and T2 synthesis

T1 was synthesized according to protocols described in the literature.[37] Briefly, for T2 synthesis, tert-butyl(((tert-butoxycarbonyl)amino) (1H-pyrazol-1-yl)methylene)carbamate (118.2 mg, 0.58 mmol) was dissolved in CH_3CN (20 mL) under Ar atmosphere. Then, 4-(5,5-dimethyl-1,3-dioxan-2-yl)benzenamine (152.2 mg, 0.49 mmol) and DIPEA (N,N-Diisopropylethylamine) (83.6 μL, 0.48 mmol) were successively added to the solution. After 18 h of stirring at 65°C, the reaction was stopped and cooled down (Figure S6).

The solvent was removed under reduced pressure, and the residue was redissolved in CH_3Cl (20 mL) and washed with HCl 10% (4 × 10 mL), and H_2O (4 × 10 mL). The organic phase was dried with $MgSO_4$ and concentrated under reduced pressure. Crude was purified by flash chromatography (Hexane:EtOAc (ethyl acetate), 9:1) affording 227.83 mg as a white solid (yield: [73.4%]. Rf = 0.3 [hexane: EtOAc, 9:1]).

Characterization of T2 was performed via H NMR (proton nuclear magnetic resonance) (400 MHz, CDCl3) δ 11.61, 10.33, 9.92, 7.82, 7.60, 7.43, 5.35, 3.74, 3.65, 1.55, 1.50, 1.28, and 0.79 (Figure S7); C NMR (carbon nuclear magnetic resonance) (101 MHz, $CDCl_3$) δ 163.55, 153.33, 137.24, 134.97, 130.92, 126.75, 121.92, 101.38, 83.71, 79.59, 30.23, 28.21, 28.11, 23.07, and 21.92 (Figure S8); and ESI-MS (electrospray ionization mass spectrometry) (CH_3Cl) m/z $(2M+ Na)^+$ 921,495 (Figure S9).

Estimation of PEI free amines

The estimation of the free amines for the functionalization of PEI was calculated based on the percentage of this specific group that reacts with fluorescamine, a dye that is only fluorescent when reacted with primary amines. To this end, first, a standard curve with ethanolamine combined with fluorescamine was created to calibrate the concentration of the conjugates by fluorescence. Ethanolamine solutions (0.15 mL) (in 5 mM HEPES, different concentrations of ethanolamine) were mixed with 0.15 mL of fluorescamine (2 mM in methanol), and the mixture was allowed to react for 30 min. The fluorescence was measured at 385 nm (excitation) and 460 ± 20 nm (emission).[38–40] The calibration line was created by plotting the emission intensity against the ethanolamine concentration and fitting these data to a straight line passing through (0,0). Then, PEI-fluorescamine conjugate was analyzed following the same procedure, and its value was extrapolated to the calibration line (Figure S10B). Lastly, calculation of the molarity of free amines concerning PEI molarity was calculated according to the following equation:

Equation 1: Calculation of PEI free amines:

$$\frac{PEI}{free\ amines}\,ratio = \frac{Real\ PEI\ concentration}{Estimated\ PEI\ concentration\ from\ extrapolation} \quad \text{(Equation 1)}$$

Conjugation of PEI with aldehyde formulations

PEI (342 mM) in acetate buffer (100 mM, pH 3.0) was reacted with 1.4 equivalents of guanidinium aldehydes (T1 and T2) and a varying amount of one hydrophobic aldehyde, either T3A or T3B. Specifically, reactions were performed with molar feed ratios of guanidinium aldehyde to hydrophobic aldehyde of 70/30 (using 0.6 equivalents of T3A or T3B), 87.5/12.5 (using 0.2 equivalents of T3A or T3B), and 93/7 (using 0.1 equivalents of T3A or T3B). The 1.4 equivalents of guanidinium aldehydes remained constant across all reactions. Briefly, 300 μL of a solution of PEI (11 mM) in acetate buffer (100 mM, pH 3.0); 17.3 μL of a solution of T1 or T2 (267 mM) in dry DMSO; and 12.7, 4.23, or 2.12 μL (for achieving 70/30, 87.5/12.5, and 93/7 ratios, respectively) of a solution of T3A or T3B (156 mM) in dry DMSO were mixed. Dry DMSO is employed as it is a hygroscopic buffer and prevents the uptake of external water from the transfectant. This mixture was shaken at 60°C for 2 h. The degree of functionalization of PEI was determined by comparing the integration of the residual aldehyde signals (T1/T2, 9.68 ppm/9.94 ppm) with the protons corresponding to the imine formation (5.14 ppm for the T1 aldehyde) and the total number of protons in the aromatic region (for T2, where the imine signal appears at 3.51 ppm and overlaps with the PEI backbone protons). As no free hydrophobic aldehydes were detected, we assumed that the reaction had proceeded to completion (0.6 equivalents) (Figures S11–S17). Polymers were used without further purification for transfection experiments.[18]

Plasmid propagation and formation of polyplexes

p*lacZ*, p*GFP*, and p*sox9* plasmids were propagated, purified, and quantified using standard methodologies. Cy3 labeling of p*lacZ* was performed using the Label IT nucleic acid labeling kit according to the manufacturer's protocol.[21]

Polyplexes were formed by mixing a specific volume of a stock solution of p*lacZ*, p*GFP*, or p*sox9* (1 μg of plasmid) with varying volumes of PEI formulations (PEI, PEIT1, PEIT1T3A, PEIT1T3B, PEIT2, PEIT2T3A, PEIT2T3B, PEIT3A, and PEIT3B) in Opti-MEM medium to achieve PEI concentrations of 5 μg/mL, 7.5 μg/mL, or 10 μg/mL and PEI/DNA estimated amino groups (N)[41] to nucleic acid anionic phosphate groups (P) ratios of 7, 14, and 21. For the estimation of the N present in the different formulations of polyplexes, the total amount of N in a PEI molecule, 21 nmol of N every μg of PEI,[41] was considered to avoid problems of different concentrations regarding different formulations of polyplexes. With respect to DNA, and as a rule, there are approximately 3 nmol of P for every μg of DNA. The mixtures were allowed to equilibrate for 30 min at room temperature (RT).

Size and zeta potential

The hydrodynamic diameter and zeta potential of both PEI formulations and polyplexes (5 μg/mL of PEI, N/P 14, in 2 mL for dynamic light scattering [DLS] and in 1 mL for electrophoretic light scattering [ELS]) were measured in aqueous medium at 25°C using DLS and ELS on a NanoBrook 90Plus Zeta instrument (Brookhaven Instruments Corporation, Holtsville, NY, USA).

Agarose gel electrophoresis

The capacity of polyplexes (prepared as described in Section 'Plasmid propagation and formation of polyplexes') to condense DNA was evaluated by agarose gel electrophoresis assay. Naked p*lacZ* (control) or polymer-complexed p*lacZ* samples were run on a 0.8% agarose gel after adding DNase I at a final concentration of 1 U DNase per 2.5 μg DNA.[16] The mixtures were incubated at 37°C for 30 min. Finally, a 7% SDS solution, commonly used as a negatively charged surfactant that disrupts the electrostatic and hydrophobic interactions between the transfectant and the DNA,[42] was added to release DNA from the polyplexes. The agarose gel was immersed in Tris-borate-EDTA buffer and exposed for 30 min to 70 V. DNA bands were stained with SYBR Green, and images were observed under a digital Chemi-Doc MP Imaging System (Bio-Rad, Madrid, Spain).

Evaluation of polyplex complexation ability

The ability of polymers to bind and complex DNA was evaluated through a fluorescence-exclusion titration assay.[43] Briefly, polyplexes were invariably prepared by mixing p*lacZ* (500 ng) with the polymers

as described in Section 'Plasmid propagation and formation of polyplexes'. Afterward, the solution was incubated for 30 min at RT, then SYBR Green (200×; 3 μL) was added, and the mixture was incubated for 10 min protected from light. Finally, 10 mM HEPES was added to obtain a total volume of 300 μL. Fluorescence measurements were performed with a Synergy HTX Plate Reader (Biotek, Winooski, VT, USA) in black 96-well plates (λ_{exc} = 485 nm and $\lambda_{em.}$ = 528 nm). The complexation efficiency (%) was expressed as relative fluorescence, normalized to the fluorescence of uncomplexed (naked) pDNA according to the following equation:[44]

Equation 2: Calculation of the percentage of normalized fluorescence emission of free DNA:

$$\textit{Fluorescence intensity}\ (\%) = \frac{F\ sample}{F\ naked\ DNA} \times 100 \qquad \text{(Equation 2)}$$

Cells isolation and culture

Bone marrow aspirates were collected from the proximal femur of patients undergoing hip arthroplasty (n = 5) and provided by the Biobanco of A Coruña from SERGAS. This study was approved by the Comité de Ética de Investigación da Coruña (accession number: 2021/425), with all patients giving informed consent. hMSCs were isolated and expanded using standard protocols.[45] Cells derived from aspirates were initially rinsed using Dulbecco's modified Eagle medium (DMEM), followed by centrifugation. The resulting cell pellet was then treated with a 1:1 solution of Red Blood Cell Lysis Buffer (Sigma-Aldrich, Madrid, Spain) in DMEM. This fraction was subsequently washed, centrifuged again, and finally resuspended in growth medium (DMEM supplemented with 10% fetal bovine serum (FBS) and 1% penicillin/streptomycin). These cells were cultured in T75 flasks under standard conditions: 37°C in a humidified environment with 5% CO_2. After the first 24 h, and then every 2–3 days, the culture medium was refreshed using growth medium. For subsequent experiments, cells were detached and replated at the required densities. iMSCs were kindly donated by Prof. S.M. Diaz-Prado.[46] Cells were grown in DMEM supplemented with 10% FBS and 1% P/S (Penicillin/Streptomycin) and kept at 37°C in a humidified atmosphere containing 5% CO_2.

hMSCs characterization

hMSCs were characterized using flow cytometry. Briefly, cells were trypsinized, washed, and incubated at 4°C for 45 min with the following antibodies: fluorescein isothiocyanate (FITC) isotype (1:50), PE isotype (1:50), PE-conjugated anti-human CD34 (1:25), FITC-conjugated anti-human CD45 (1:25), PE-conjugated anti-human CD73 (1:25), FITC-conjugated anti-human CD90 (1:25), and FITC-conjugated anti-human CD105 (1:5). After this, cells were washed, resuspended, and transferred to polypropylene tubes (NUNC, VWR International, Radnor, PA, USA). Acquisition was performed using a CytoFlex cytometer (Beckman Coulter Life Sciences, Madrid, Spain), with data analyzed via CytExpert software (Beckman Coulter Life Sciences). A minimum of 10^4 cell events were acquired and analyzed per assay.

Evaluation of gene transfer efficiency using polymer formulations

iMSCs and hMSCs were seeded in 96-well plates at an initial density of 10^4 cells/well and allowed to adhere for 24 h at 37°C before the experiments. Cells were exposed to p*lacZ* or pGFP polyplexes by adding a solution of a non-supplemented medium (100 μL) with the respective concentration of polyplexes (250 ng of DNA) to each well. Negative and positive controls included untreated hMSCs and cells transfected with unmodified PEI or LPF (1 μL/well). After transfection, cells were incubated for 24 h at 37°C and 5% CO_2 before performing the different assays. Each condition was assessed by quadruplicate.

Transfection efficiency with p*lacZ* polyplexes was determined using the β-Glo reagent. The β-Glo reagent facilitates a coupled enzymatic reaction using the luciferin-galactoside substrate 6-O-β-galactopyranosylluciferin. β-Galactosidase from transfected cells cleaves this substrate, yielding luciferin and galactose. Subsequently, the luciferin reacts with firefly luciferase, producing light. Luminescence was measured in white polystyrene 96-well plates using a Synergy HTX Plate Reader (Biotek, Winooski, VT, USA), and the β-galactosidase activity measured in the sample was normalized to that assessed in a PEI control to obtain the fold increase in β-galactosidase activity.[47]

Equation 3: Calculation of gene transfer efficiency by β-galactosidase activity fold increase:

$$\beta - \textit{galactosidase activity fold increase} = \frac{RLU\ sample}{RLU\ PEI\ control} \qquad \text{(Equation 3)}$$

Transfection efficiency with p*GFP* polyplexes was quantified by assessing the GFP-positive events quantified in the sample, normalized to those measured in a PEI control (GFP activity fold increase). In brief, cells were washed, resuspended, and transferred to polypropylene tubes (NUNC, VWR International, Radnor, PA, USA). The acquisition was performed using a CytoFlex cytometer (Beckman Coulter Life Sciences, Madrid, Spain) by checking the positive events registered while employing the FITC filter (B525-A, 525/50 nm), with data analyzed via CytExpert software (Beckman Coulter Life Sciences, Madrid, Spain). A minimum of 10^4 cell events were acquired and analyzed per assay.

Equation 4: Calculation of gene transfer efficiency by GFP activity fold increase:

$$\textit{GFP activity fold increase} = \frac{GFPpositive\ events\ in\ sample\ (\%)}{GFPpositive\ events\ in\ PEI\ control\ (\%)} \qquad \text{(Equation 4)}$$

Assessment of cell viability using polymer formulations

The viability of iMSCs and hMSCs monolayers was monitored at 24 h post-transfection using the commercial reagent Cell Counting

Kit-8 (MedChemExpress, NJ, US). This kit uses a water-soluble tetrazolium salt, WST-8, which is reduced to a water-soluble formazan dye by the metabolic activity of viable cells. The amount of formazan produced, measured via absorbance, is directly proportional to the number of living cells. Absorbance (A) at 450 nm was measured using a Synergy HTX Plate Reader and the percentage of cell viability (%) was calculated using the following equation:[48]

Equation 5: Calculation of cell viability percentage by normalization of absorbance values:

$$Cell\ viability\ (\%) = \frac{A\ sample}{A\ negative\ control} \times 100 \qquad \text{(Equation 5)}$$

The viability of hMSCs transfected with pGFP polyplexes was evaluated by staining the cells with 200 μL of propidium iodide (PI, 10 μg/mL) (Invitrogen, Madrid, Spain) before quantifying the events by flow cytometry. Cell viability (%) was calculated by subtracting the percentage of PI-positive cells to the total number of cells counted:

Equation 6: Calculation of cell viability percentage by normalization of the total number of events:

$$Cell\ viability\ (\%) = \frac{Total\ number\ of\ events\ -\ PI\ positive\ events}{Total\ number\ of\ events} \times 100 \qquad \text{(Equation 6)}$$

Internalization mechanism of PEIT2T3A polyplexes

To study polyplexes internalization mechanisms in hMSCs, a pre-treatment with various endocytosis inhibitors was performed before transfection experiments.[49] Cells (10^4/well) were plated in 96-well plates and incubated for 24 h at 37°C before experiments. Pre-treatment with inhibitors involved chlorpromazine (30 μM, 1 h), genistein (200 μM, 1 h), methyl-β-cyclodextrin (2 mM, 10 min), and amiloride (5 mM, 10 min) for the respective inhibition of clathrin-mediated endocytosis, caveolae-mediated endocytosis, clathrin- and caveolae-dependent endocytosis, and macropinocytosis. After pre-treatment, PEIT2T3A polyplexes (250 ng plasmid/well; cationic/hydrophobic aldehyde ratio of 70/30) were added and incubated for 4 h. Control conditions included cells transfected with polyplexes without inhibitor pre-treatment. β-Galactosidase activity and cell viability quantification were assessed following the same protocols described in Sections 'Evaluation of Gene Transfer Efficiency Using Polymer Formulations' and 'Assessment of Cell Viability Using Polymer Formulations'.

Visualization of PEIT2T3A polyplexes uptake was also monitored through confocal microscopy. hMSCs (10,000 cells/well) were seeded in 8-well μ-chamber slides and incubated for 2 h with Cy3-labeled polyplexes with either AlexaFluor488-Cholera Toxin (10 μg/mL), a marker for caveolae/lipid raft endocytosis, or AlexaFluor488-Transferrin (50 μg/mL), a marker for clathrin-mediated endocytosis.[34] After incubation, cells were washed with PBS fixed with a 4% paraformaldehyde solution, and cell nuclei were stained with Hoechst 33342. Untreated cells were assessed in parallel as a negative control group. Image acquisition was carried out with an AR1 confocal microscope (Nikon, Tokyo, Japan).

Chondrogenic differentiation of hMSCs using PEIT2T3A polyplexes

hMSCs (2×10^5 cells) were centrifuged to form cell aggregates or pellets and cultured in a defined CT (formulated by adding penicillin-streptomycin, dexamethasone, ascorbic acid, pyruvate, insulin-transferrin-selenium A (ITS), and TGF-β3 to non-supplemented DMEM) under static conditions at 37°C for 21 days).[45,50] Prior to the addition of CT, hMSCs aggregates were transfected with PEIT2T3A/p*sox9* polyplex formulation (1 μg plasmid; cationic/hydrophobic aldehyde ratio of 70/30), prepared according to the method depicted in Section 'Evaluation of Gene Transfer Efficiency Using Polymer Formulations'. Control groups consisted of aggregates transfected with the same dose of plasmid complexed with unmodified PEI (positive control) and untreated aggregates cultured in CT (negative control).

Histological and immunohistochemical analysis

hMSC aggregates were harvested and subjected to standard paraffin embedding procedures, including fixation and dehydration.[45,50] Sections (4 μm) were stained with toluidine blue (matrix proteoglycans) and H&E (cell nuclei and cytoplasm).[50] Immunohistochemistry was performed using specific primary antibodies (SOX9 and types -I, -II, and -X collagen), anti-mouse biotinylated secondary antibodies, and the ABC method with diaminobenzidine as chromogen.[51,52] Controls of non-transfected and PEI-transfected cells were included. An Olympus CKX53 light microscope was used for sample examination.

Histomorphometry

Quantitation of cell density (cells/mm^2) from H&E-stained sections, along with toluidine blue and immunohistochemical staining intensities for SOX9 and collagen types I, II, and X, was performed using CellSens and ImageJ software. Measurements were taken from three randomly selected standardized areas at 20× magnification for each experimental condition and replicate. The staining intensities were expressed as pixels per standardized area.

Total RNA extraction and real-time RT-PCR analyses

Total RNA was extracted from hMSCs aggregates using a RNeasy Protect Mini Kit, with on-column DNase treatment to remove genomic DNA. Reverse transcription with the resulting RNA eluate (8 μL) was performed with the 1st Strand cDNA Synthesis kit (AMV). cDNA (2.5 μL at 125 ng/μL) was then amplified by qPCR using Power Up SYBR Green Master Mix on a QuantStudio3 qPCR instrument (Thermo Fisher Scientific, Madrid, Spain). The qPCR protocol consisted of a 2-min hold at 50°C, a 2-min denaturation at 95°C C, and 40 cycles of amplification: 95°C for 15 s (denaturation) and 60°C for 1 min (annealing/extension). A melt curve analysis was

also performed using the following steps: 95°C for 5 s, 65°C for 10 s with a temperature gradient of 1.6°C/second, and 95°C for 1 s with a temperature gradient of 0.5°C/second. Primers (Life Technologies Thermo Fisher Scientific, Madrid, Spain) were used at a final concentration of 300 nM. The target genes and primer sequences were as follows: aggrecan, which served as a chondrogenic marker for proteoglycans (ACAN, forward: 5′-GAGATGGAGGGTGAGGTC-3′, reverse: 5′-ACGCTGCCTCGGGCTTC-3′); SOX9, as an early chondrogenic transcription factor (forward: 5′-ACACACAGCTCACTC GACCTTG-3′); COLII, as a chondrogenic and matrix marker (COLII, forward: 5′-GGACTTTTCTCCCCTCTCT-3′, reverse: 5′-G ACCCGAAGGTCTTACAGGA-3′); type I collagen, as an osteogenic marker (COLI, forward: 5′-ACGTCCTGGTGAAGTTGGTC-3′, reverse: 5′-ACCAGGGAAGCCTCTCTCTC-3′); type X collagen, as a marker of hypertrophy (COLX, forward: 5′-CCCTCTTGTTA GTGCCAACC-3′, reverse: 5′-AGATTCCAGTCCTTGGGTCA-3′); and GAPDH, which was used as a housekeeping gene (forward: 5′-GAAGGTGAAGGTCGGAGTC-3′, reverse: 5′-GAAGATGGTG ATGGGATTTC-3′). Gene expression was quantified using QuantStudio software. Threshold cycle (Ct) values for each gene were normalized to GAPDH expression, and fold inductions relative to untreated control aggregates were calculated using the $2^{-\Delta\Delta Ct}$ method.[45,50,53]

Biochemical analysis

Proteoglycan content in hMSC aggregates was determined with DMMB dye, using chondroitin sulfate as standard. hMSCs were digested with papain (125 μg/mL, pH 6.5) at 60°C for 1 h[50] before biochemical assays. Proteoglycan amounts of each sample were normalized to either total protein (BCA assay) or DNA (Hoechst 33342 assay) contents.[54] A Synergy HTX Plate Reader was used for all quantifications.

Statistical analysis

Results were expressed as mean ± standard deviation (SD) of three or four technical replicates. Statistical significance ($p \leq 0.05$) was determined using GraphPad Prism 9, employing one-way ANOVA or Student's t test for parametric data, and Kruskal-Wallis, Multiple Range, or Mann-Whitney U tests for non-parametric data as appropriate.

DATA AND CODE AVAILABILITY

All data reported in this paper will be shared upon request.

ACKNOWLEDGMENTS

A.R.-R. thanks the Ministerio de Ciencia e Innovación for her Ramon y Cajar Fellowship (RYC2018-025617-I). J.L.-S. thanks MICINN for her pre-doctoral fellowship (FPU20/06176). A.R.-B. thanks MICINN for her pre-doctoral fellowship (PRE2022-104070). P.F.-T. thanks the Spanish Ministerio de Educación, Cultura y Deporte for a Beatriz Galindo Award (BG20/00213). Xunta de Galicia supported this work (ED431F 2021/10 and ED431B 2023/60). This work is part of the project PID2021-128461OB-I00, funded by MCIN/AEI/10.13039/501100011033/FEDER, UE. We also thank Biobanco de A Coruña from SERGAS for providing biological samples.

AUTHOR CONTRIBUTIONS

D.M.-B., A.R.-R., and P.F.-T. conceived and designed the experiments. A.R.-B. prepared T1 and T2 and PEI formulations. J.L.-S. developed cell and DNA methods and assays. D.M.-B. and J.F.-L. performed cytometry. D.M.-B. and I.L.-C. performed histology. D.M.-B. performed all other experiments. A.R.-R. and P.F.-T. secured funding. D.M.-B., A.R.-R., and P.F.-T. analyzed the data and wrote the paper, with all other authors contributing to the final version of the manuscript.

DECLARATION OF INTERESTS

The authors declare no competing interests.

SUPPLEMENTAL INFORMATION

Supplemental information can be found online at https://doi.org/10.1016/j.omtn.2025.102551.

REFERENCES

1. Ma, C.-C., Wang, Z.-L., Xu, T., He, Z.-Y., and Wei, Y.-Q. (2020). The approved gene therapy drugs worldwide: from 1998 to 2019. Biotechnol. Adv. *40*, 107502. https://doi.org/10.1016/j.biotechadv.2019.107502.
2. Ansari, A.S., Santerre, P.J., and Uludağ, H. (2017). Biomaterials for polynucleotide delivery to anchorage-independent cells. J. Mater. Chem. B *5*, 7238–7261. https://doi.org/10.1039/c7tb01833a.
3. Lostalé-Seijo, I., and Montenegro, J. (2018). Synthetic materials at the forefront of gene delivery. Nat. Rev. Chem *2*, 258–277. https://doi.org/10.1038/s41570-018-0039-1.
4. Jere, D., Jiang, H.L., Arote, R., Kim, Y.K., Choi, Y.J., Cho, M.H., Akaike, T., and Cho, C.S. (2009). Degradable polyethylenimines as DNA and small interfering RNA carriers. Expert Opin. Drug Deliv. *6*, 827–834. https://doi.org/10.1517/17425240903029183.
5. Pandey, A.P., and Sawant, K.K. (2016). Polyethylenimine: A versatile, multifunctional non-viral vector for nucleic acid delivery. Mater. Sci. Eng.: C *68*, 904–918. https://doi.org/10.1016/j.msec.2016.07.066.
6. Acri, T.M., Laird, N.Z., Geary, S.M., Salem, A.K., and Shin, K. (2019). Effects of calcium concentration on nonviral gene delivery to bone marrow-derived stem cells. J. Tissue Eng. Regen. Med. *13*, 2256–2265. https://doi.org/10.1002/term.2971.
7. Mintzer, M.A., and Simanek, E.E. (2009). Nonviral Vectors for Gene Delivery. Chem. Rev. *109*, 259–302. https://doi.org/10.1021/cr800409e.
8. Nimesh, S., and Chandra, R. (2008). Guanidinium-grafted polyethylenimine: An efficient transfecting agent for mammalian cells. Eur. J. Pharm. Biopharm. *68*, 647–655. https://doi.org/10.1016/j.ejpb.2007.09.008.
9. Vazdar, M., Heyda, J., Mason, P.E., Tesei, G., Allolio, C., Lund, M., and Jungwirth, P. (2018). Arginine "Magic": Guanidinium Like-Charge Ion Pairing from Aqueous Salts to Cell Penetrating Peptides. Acc. Chem. Res. *51*, 1455–1464. https://doi.org/10.1021/acs.accounts.8b00098.
10. Yan, X., Zhang, Y., Zhang, H., Wang, P.G., Chu, X., and Wang, X. (2014). Amphiphilic polyethylenimine (PEI) as highly efficient non-viral gene carrier. Org. Biomol. Chem. *12*, 1975–1982. https://doi.org/10.1039/c3ob42279h.
11. Gabrielson, N.P., and Pack, D.W. (2006). Acetylation of Polyethylenimine Enhances Gene Delivery via Weakened Polymer/DNA Interactions. Biomacromolecules *7*, 2427–2435. https://doi.org/10.1021/bm060300u.
12. Doody, A.M., Korley, J.N., Dang, K.P., Zawaneh, P.N., and Putnam, D. (2006). Characterizing the structure/function parameter space of hydrocarbon-conjugated branched polyethylenimine for DNA delivery in vitro. J. Control. Release *116*, 227–237. https://doi.org/10.1016/j.jconrel.2006.07.019.
13. Oskuee, R.K., Dehshahri, A., Shier, W.T., and Ramezani, M. (2009). Alkylcarboxylate grafting to polyethylenimine: a simple approach to producing a DNA nanocarrier with low toxicity. J. Gene Med. *11*, 921–932. https://doi.org/10.1002/jgm.1374.
14. Zhang, Q.-F., Luan, C.-R., Yin, D.-X., Zhang, J., Liu, Y.-H., Peng, Q., Xu, Y., and Yu, X.-Q. (2015). Amino Acid-Modified Polyethylenimines with Enhanced Gene Delivery Efficiency and Biocompatibility. Polymers *7*, 2316–2331. https://doi.org/10.3390/polym7111516.
15. Hamann, A., Nguyen, A., and Pannier, A.K. (2019). Nucleic acid delivery to mesenchymal stem cells: a review of nonviral methods and applications. J. Biol. Eng. *13*, 7. https://doi.org/10.1186/s13036-019-0140-0.

16. Attia, N., Mashal, M., Grijalvo, S., Eritja, R., Zárate, J., Puras, G., and Pedraz, J.L. (2018). Stem cell-based gene delivery mediated by cationic niosomes for bone regeneration. Nanomedicine. *14*, 521–531. https://doi.org/10.1016/j.nano.2017.11.005.

17. Pawitan, J.A., Bui, T.A., Mubarok, W., Antarianto, R.D., Nurhayati, R.W., Dilogo, I. H., and Oceandy, D. (2020). Enhancement of the Therapeutic Capacity of Mesenchymal Stem Cells by Genetic Modification: A Systematic Review. Front. Cell Dev. Biol. *8*, 587776. https://doi.org/10.3389/fcell.2020.587776.

18. Priegue, J.M., Lostalé-Seijo, I., Crisan, D., Granja, J.R., Fernández-Trillo, F., and Montenegro, J. (2018). Different-Length Hydrazone Activated Polymers for Plasmid DNA Condensation and Cellular Transfection. Biomacromolecules *19*, 2638–2649. https://doi.org/10.1021/acs.biomac.8b00252.

19. Juanes, M., Creese, O., Fernández-Trillo, P., and Montenegro, J. (2019). Messenger RNA delivery by hydrazone-activated polymers. MedChemComm *10*, 1138–1144. https://doi.org/10.1039/c9md00231f.

20. Crisan, D.N., Creese, O., Ball, R., Brioso, J.L., Martyn, B., Montenegro, J., and Fernandez-Trillo, F. (2017). Poly(acryloyl hydrazide), a versatile scaffold for the preparation of functional polymers: synthesis and post-polymerisation modification. Polym. Chem. *8*, 4576–4584. https://doi.org/10.1039/c7py00535k.

21. Carballo-Pedrares, N., Sanjurjo-Rodriguez, C., Señarís, J., Díaz-Prado, S., and Rey-Rico, A. (2022). Chondrogenic Differentiation of Human Mesenchymal Stem Cells via SOX9 Delivery in Cationic Niosomes. Pharmaceutics *14*, 2327. https://doi.org/10.3390/pharmaceutics14112327.

22. Akiyama, H., Lyons, J.P., Mori-Akiyama, Y., Yang, X., Zhang, R., Zhang, Z., Deng, J. M., Taketo, M.M., Nakamura, T., Behringer, R.R., et al. (2004). Interactions between Sox9 and β-catenin control chondrocyte differentiation. Genes Dev. *18*, 1072–1087. https://doi.org/10.1101/gad.1171104.

23. He, Y., Cheng, G., Xie, L., Nie, Y., He, B., and Gu, Z. (2013). Polyethyleneimine/DNA polyplexes with reduction-sensitive hyaluronic acid derivatives shielding for targeted gene delivery. Biomaterials *34*, 1235–1245. https://doi.org/10.1016/j.biomaterials.2012.09.049.

24. Ogris, M., Steinlein, P., Kursa, M., Mechtler, K., Kircheis, R., and Wagner, E. (1998). The size of DNA/transferrin-PEI complexes is an important factor for gene expression in cultured cells. Gene Ther. *5*, 1425–1433. https://doi.org/10.1038/sj.gt.3300745.

25. Godbey, W.T., Wu, K.K., and Mikos, A.G. (1999). Size matters: Molecular weight affects the efficiency of poly(ethylenimine) as a gene delivery vehicle. J. Biomed. Mater. Res. *45*, 268–275. https://doi.org/10.1002/(sici)1097-4636(19990605)45:3<268::aid-jbm15>3.0.co;2-q.

26. Chong, Z.X., Yeap, S.K., and Ho, W.Y. (2021). Transfection types, methods and strategies: a technical review. PeerJ *9*, e11165. https://doi.org/10.7717/peerj.11165.

27. Gresch, O., and Altrogge, L. (2012). Protein Expression in Mammalian Cells, Methods and Protocols. Methods Mol. Biol. *801*, 65–74. https://doi.org/10.1007/978-1-61779-352-3_5.

28. Patnaik, S., Arif, M., Pathak, A., Kurupati, R., Singh, Y., and Gupta, K.C. (2010). Cross-linked polyethylenimine-hexametaphosphate nanoparticles to deliver nucleic acids therapeutics. Nanomedicine. *6*, 344–354. https://doi.org/10.1016/j.nano.2009.07.007.

29. Oh, Y.-K., Suh, D., Kim, J.M., Choi, H.-G., Shin, K., and Ko, J.J. (2002). Polyethylenimine-mediated cellular uptake, nucleus trafficking and expression of cytokine plasmid DNA. Gene Ther. *9*, 1627–1632. https://doi.org/10.1038/sj.gt.3301735.

30. Forrest, M.L., Koerber, J.T., and Pack, D.W. (2003). A Degradable Polyethylenimine Derivative with Low Toxicity for Highly Efficient Gene Delivery. Bioconjug. Chem. *14*, 934–940. https://doi.org/10.1021/bc034014g.

31. Lim, Y.b., Kim, S.m., Suh, H., and Park, J.s. (2002). Biodegradable, Endosome Disruptive, and Cationic Network-type Polymer as a Highly Efficient and Nontoxic Gene Delivery Carrier. Bioconjug. Chem. *13*, 952–957. https://doi.org/10.1021/bc025541n.

32. Fischer, D., Bieber, T., Li, Y., Elsässer, H.-P., and Kissel, T. (1999). A Novel Non-Viral Vector for DNA Delivery Based on Low Molecular Weight, Branched Polyethylenimine: Effect of Molecular Weight on Transfection Efficiency and Cytotoxicity. Pharm. Res. *16*, 1273–1279. https://doi.org/10.1023/a:1014861900478.

33. Gonzalez-Fernandez, T., Sathy, B.N., Hobbs, C., Cunniffe, G.M., McCarthy, H.O., Dunne, N.J., Nicolosi, V., O'Brien, F.J., and Kelly, D.J. (2017). Mesenchymal stem cell fate following non-viral gene transfection strongly depends on the choice of delivery vector. Acta Biomater. *55*, 226–238. https://doi.org/10.1016/j.actbio.2017.03.044.

34. Puras, G., Mashal, M., Zárate, J., Agirre, M., Ojeda, E., Grijalvo, S., Eritja, R., Diaz-Tahoces, A., Martínez Navarrete, G., Avilés-Trigueros, M., et al. (2014). A novel cationic niosome formulation for gene delivery to the retina. J. Control. Release *174*, 27–36. https://doi.org/10.1016/j.jconrel.2013.11.004.

35. Rehman, Z.u., Zuhorn, I.S., and Hoekstra, D. (2013). How cationic lipids transfer nucleic acids into cells and across cellular membranes: Recent advances. J. Control. Release *166*, 46–56. https://doi.org/10.1016/j.jconrel.2012.12.014.

36. Kiss, A.L., Turi, Á., Müller, N., Kántor, O., and Botos, E. (2002). Caveolae and caveolin isoforms in rat peritoneal macrophages. Micron *33*, 75–93. https://doi.org/10.1016/s0968-4328(00)00100-1.

37. Priegue, J.M., Crisan, D.N., Martínez-Costas, J., Granja, J.R., Fernandez-Trillo, F., and Montenegro, J. (2016). In Situ Functionalized Polymers for siRNA Delivery. Angew. Chem. Int. Ed. *55*, 7492–7495. https://doi.org/10.1002/anie.201601441.

38. Udenfriend, S., Stein, S., Böhlen, P., Dairman, W., Leimgruber, W., and Weigele, M. (1972). Fluorescamine: A Reagent for Assay of Amino Acids, Peptides, Proteins, and Primary Amines in the Picomole Range. Science *178*, 871–872. https://doi.org/10.1126/science.178.4063.871.

39. (PDF) Fluorimetric Quantitation of Protein using the Reactive Compound Fluorescamine https://www.researchgate.net/publication/232755057_Fluorimetric_Quantitation_of_Protein_using_the_Reactive_Compound_Fluorescamine.

40. Çelen, T., Anumudu, C., Miri, T., Onyeaka, H., and Fernandez-Trillo, P. (2023). Nisin:Carboxymethylcellulose polyion complex (PIC) nanoparticles. Preparation and antimicrobial activity. Carbohydr. Polym. *317*, 121032. https://doi.org/10.1016/j.carbpol.2023.121032.

41. Ramos-Murillo, A.I., Rodríguez, E., Beltrán, K., Ricaurte, C., Camacho, B., Salguero, G., and Godoy-Silva, R.D. (2020). Efficient Non-Viral Gene Modification of Mesenchymal Stromal Cells from Umbilical Cord Wharton's Jelly with Polyethylenimine. Pharmaceutics *12*, 896. https://doi.org/10.3390/pharmaceutics12090896.

42. López-Seijas, J., Miranda-Balbuena, D., Iglesias-Fente, A., Sacristán-Santos, M., Carballo-Pedrares, N., Arufe, M.C., Rey-Rico, A., and Fafián-Labora, J. (2023). Development of new non-viral systems for genetic modification of senescent cells. Mol. Ther. Nucleic Acids *32*, 302–317. https://doi.org/10.1016/j.omtn.2023.03.010.

43. Ayyadevara, V.S.S.A., and Roh, K.-H. (2020). Calcium enhances polyplex-mediated transfection efficiency of plasmid DNA in Jurkat cells. Drug Deliv. *27*, 805–815. https://doi.org/10.1080/10717544.2020.1770371.

44. Bono, N., Ponti, F., Mantovani, D., and Candiani, G. (2020). Non-Viral in Vitro Gene Delivery: It is Now Time to Set the Bar. Pharmaceutics *12*, 183. https://doi.org/10.3390/pharmaceutics12020183.

45. Rey-Rico, A., Venkatesan, J.K., Frisch, J., Rial-Hermida, I., Schmitt, G., Concheiro, A., Madry, H., Alvarez-Lorenzo, C., and Cucchiarini, M. (2015). PEO–PPO–PEO micelles as effective rAAV-mediated gene delivery systems to target human mesenchymal stem cells without altering their differentiation potency. Acta Biomater. *27*, 42–52. https://doi.org/10.1016/j.actbio.2015.08.046.

46. Piñeiro-Ramil, M., Castro-Viñuelas, R., Sanjurjo-Rodríguez, C., Rodríguez-Fernández, S., Hermida-Gómez, T., Blanco-García, F.J., Fuentes-Boquete, I., and Díaz-Prado, S. (2020). Immortalizing Mesenchymal Stromal Cells from Aged Donors While Keeping Their Essential Features. Stem Cells Int. *2020*, 5726947. https://doi.org/10.1155/2020/5726947.

47. Rey-Rico, A., Frisch, J., Venkatesan, J.K., Schmitt, G., Rial-Hermida, I., Taboada, P., Concheiro, A., Madry, H., Alvarez-Lorenzo, C., and Cucchiarini, M. (2016). PEO-PPO-PEO Carriers for rAAV-Mediated Transduction of Human Articular Chondrocytes in Vitro and in a Human Osteochondral Defect Model. ACS Appl. Mater. Interfaces *8*, 20600–20613. https://doi.org/10.1021/acsami.6b06509.

48. Alvarez-Rivera, F., Rey-Rico, A., Venkatesan, J.K., Diaz-Gomez, L., Cucchiarini, M., Concheiro, A., and Alvarez-Lorenzo, C. (2020). Controlled Release of rAAV Vectors from APMA-Functionalized Contact Lenses for Corneal Gene Therapy. Pharmaceutics *12*, 335. https://doi.org/10.3390/pharmaceutics12040335.

49. Vanegas Sáenz, J.R., Tenkumo, T., Kamano, Y., Egusa, H., and Sasaki, K. (2017). Amiloride-enhanced gene transfection of octa-arginine functionalized calcium phosphate nanoparticles. PLoS One *12*, e0188347. https://doi.org/10.1371/journal.pone.0188347.

50. Rey-Rico, A., Venkatesan, J.K., Frisch, J., Schmitt, G., Monge-Marcet, A., Lopez-Chicon, P., Mata, A., Semino, C., Madry, H., and Cucchiarini, M. (2015). Effective and durable genetic modification of human mesenchymal stem cells via controlled release of rAAV vectors from self-assembling peptide hydrogels with a maintained differentiation potency. Acta Biomater. *18*, 118–127. https://doi.org/10.1016/j.actbio.2015.02.013.

51. Meng, W., Rey-Rico, A., Claudel, M., Schmitt, G., Speicher-Mentges, S., Pons, F., Lebeau, L., Venkatesan, J.K., and Cucchiarini, M. (2020). rAAV-Mediated Overexpression of SOX9 and TGF-β via Carbon Dot-Guided Vector Delivery Enhances the Biological Activities in Human Bone Marrow-Derived Mesenchymal Stromal Cells. Nanomaterials *10*, 855. https://doi.org/10.3390/nano10050855.

52. Rey-Rico, A., Venkatesan, J.K., Schmitt, G., Speicher-Mentges, S., Madry, H., and Cucchiarini, M. (2018). Effective Remodelling of Human Osteoarthritic Cartilage by sox9 Gene Transfer and Overexpression upon Delivery of rAAV Vectors in Polymeric Micelles. Mol. Pharm. *15*, 2816–2826. https://doi.org/10.1021/acs.molpharmaceut.8b00331.

53. Livak, K.J., and Schmittgen, T.D. (2001). Analysis of Relative Gene Expression Data Using Real-Time Quantitative PCR and the $2^{-\Delta\Delta CT}$ Method. Methods *25*, 402–408. https://doi.org/10.1006/meth.2001.1262.

54. Rey-Rico, A., Venkatesan, J.K., Sohier, J., Moroni, L., Cucchiarini, M., and Madry, H. (2015). Adapted chondrogenic differentiation of human mesenchymal stem cells via controlled release of TGF-β1 from poly(ethylene oxide)–terephtalate/poly(butylene terepthalate) multiblock scaffolds. J. Biomed. Mater. Res., Part A *103*, 371–383. https://doi.org/10.1002/jbm.a.35181.

OMTN, Volume *36*

Supplemental information

Novel PEI-aldehyde conjugates for gene delivery: Promoting chondrogenic differentiation in human mesenchymal stem cells

Diego Miranda-Balbuena, Alba Ramil-Bouzas, Naiara Doldán-Mata, Junquera López-Seijas, Juan Fafián-Labora, Ibán Lamas-Criado, Jose-Ramón Caeiro-Rey, Paco Fernández-Trillo, and Ana Rey-Rico

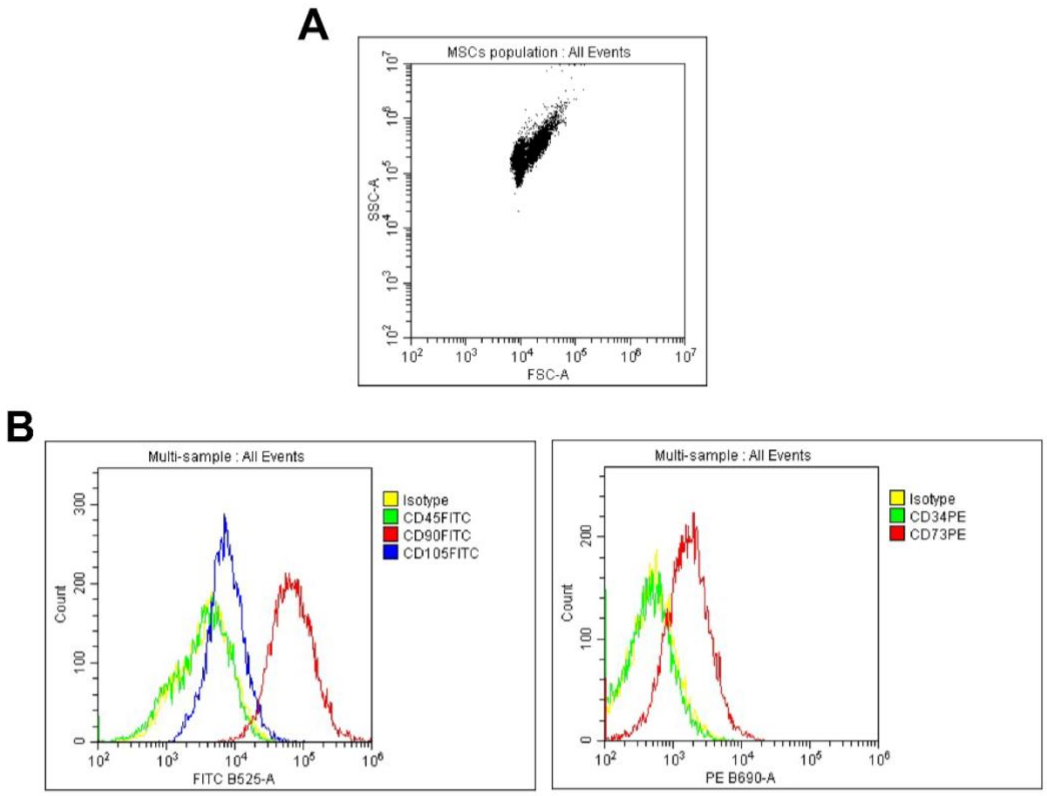

***Figure S1. Representative images for the analysis of hMSCs surface markers**. Flow cytometry analysis of hMSCs markers CD90, CD73, and CD105 and hematopoietic markers CD45, and CD34, compared with their corresponding isotype FITC or PE as negative control.*

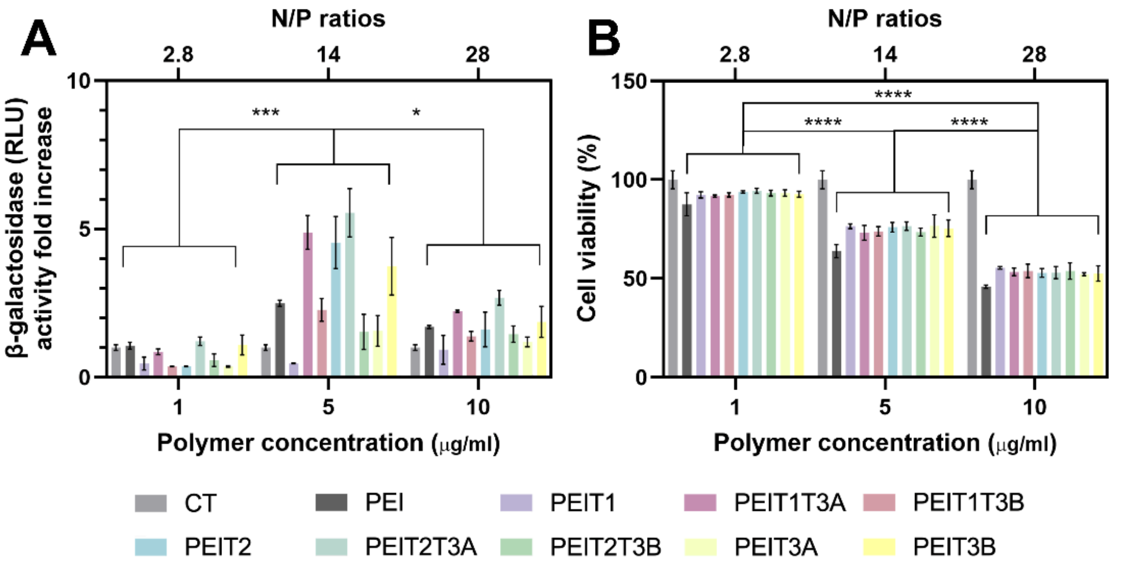

***Figure S2. Effect of broad transfectant concentrations on transfection efficiency and cell viability.** (A) β-galactosidase activity and (B) cell viability after transfection with different polyplexes (PEI, PEIT1, PEIT1T3A, PEIT1T3B, PEIT2, PEIT2T3A, PEIT2T3B, PEIT3A, and PEIT3B) (placZ) at various concentrations (1 μg/ml, 5 μg/ml, and 10 μg/ml) and N/P ratios (2.8, 14, and 28) in hMSCs. Cationic/hydrophobic aldehyde ratio = 70/30, and [placZ] = 2.5 μg/mL. * depicts $p < 0.05$, *** $p < 0.001$, and **** $p < 0.0001$ when compared denoted groups. Data is expressed as mean of quadruplicates; error bars indicate standard deviation.*

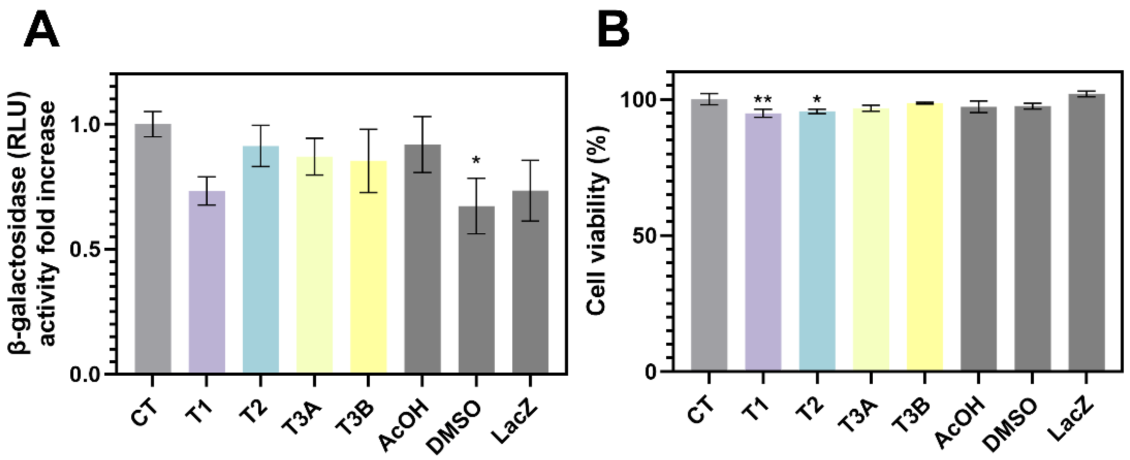

Figure S3. Transfection efficiency and cell viability of negative controls. *(A) β-galactosidase activity and (B) cell viability after transfection with different control conditions (aldehydes: T1, T2, T3A, and T3B, acetic acid (AcOH), DMSO, and plasmid placZ alone) in hMSCs. [placZ] = 2.5 μg/ml. * depicts p < 0.05, and ** p < 0.01 when compared denoted groups. Data is expressed as mean of quadruplicates; error bars indicate standard deviation.*

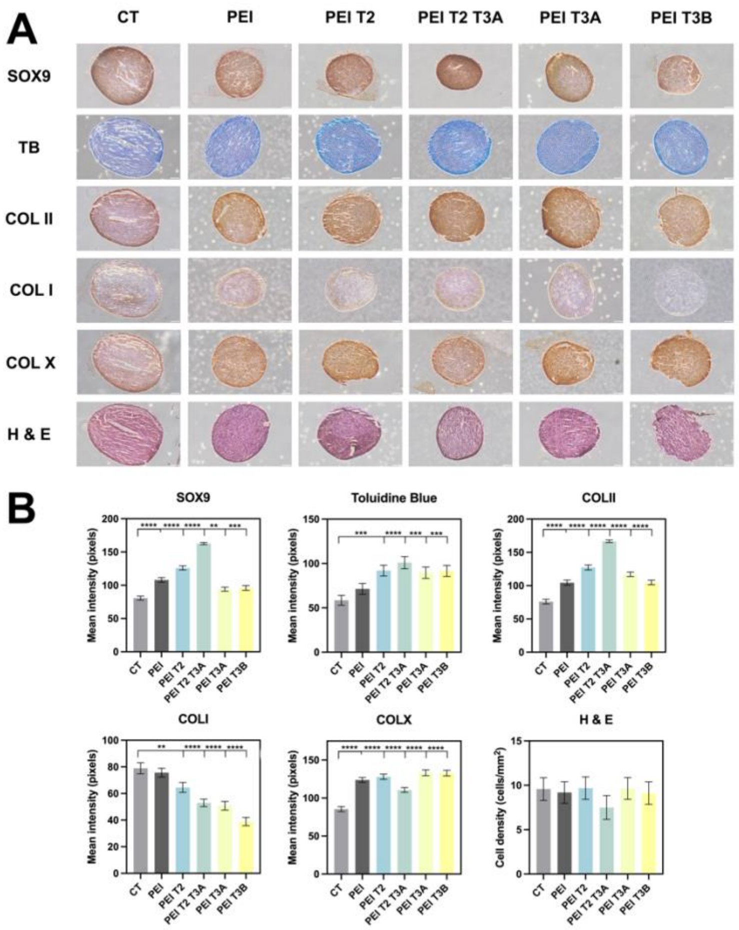

Figure S4. Immunohistochemical and histological analyses of chondrogenesis for all conditions. *Immunohistochemical and histological analyses of hMSC aggregates cultured in chondrogenic medium (CT; negative control) and transfected with psox9 via PEI, PEIT2, PEIT2T3E, PÈIT3E, or PEIT3F. Samples were kept in culture for 21 days and processed for (A) Immunodetection of SOX9, toluidine blue (TB), type-II collagen (COLII), type-I collagen (COLI), type-X collagen (COLX), and Hematoxylin/Eosin (H&E) (all representative images; magnification 10X; scale bar 100 μm). (B) Histomorphometric analyses of the previously mentioned conditions. Cationic/hydrophobic aldehyde ratio = 70/30, [PEI] = 5 μg/ml, and [placZ]*

= 5 µg/ml. ** depicts p < 0.01, *** p < 0.001, and **** p < 0.0001 when compared with denoted groups. Data is expressed as mean of triplicates; error bars indicate standard deviation.

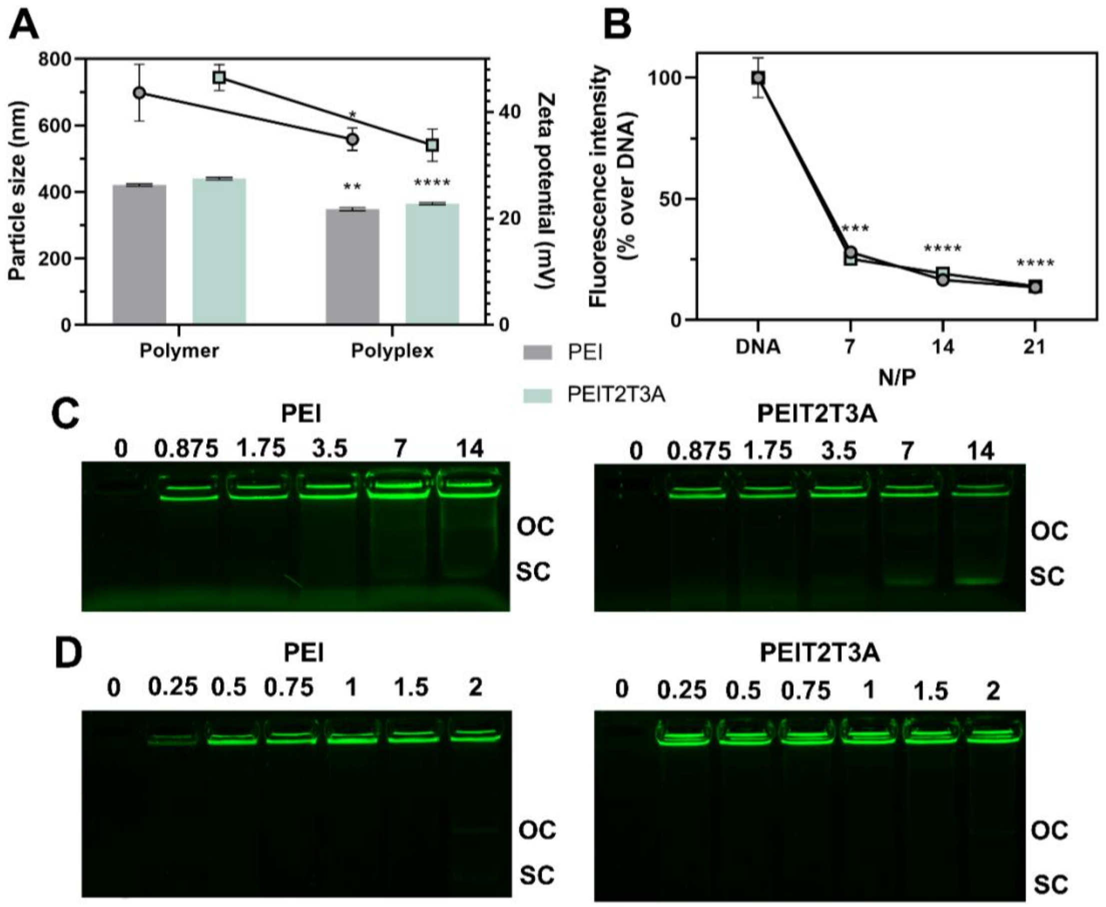

Figure S5. Unmodified PEI and PEIT2T3A characterization. *(A) Particle size (bars) and zeta potential (dots) of PEI and PEIT2T3E polymers and polyplexes (placZ). Cationic/hydrophobic aldehyde ratio = 70/30, [PEI] = 5 µg/ml, and [placZ] = 5 µg/ml (B) DNA placZ complexation efficiency of PEI and PEIT2T3E polyplexes formed at different concentrations. Cationic/hydrophobic aldehyde ratio = 70/30 and [PEI] = 5 µg/ml. (C) DNase protection ability and SDS-induced release visualized by agarose electrophoresis of PEI and PEIT2T3E formed at N/P ratios of 0.875/1, 1.75/1, 3.5/1, 7/1, and 14/1 (placZ). (D) DNase protection ability and SDS-induced release visualized by agarose electrophoresis of PEI and PEIT2T3E formed at N/P ratios of 0.25/1, 0.5/1, 0.75/1, 1/1, 1.5/1, and 2/1 (placZ). Cationic/hydrophobic aldehyde ratio = 70/30 and [PEI] = 5 µg/ml. 0: naked placZ; OC: open circular; SC: supercoiled * depicts p < 0.05, ** p < 0.01, *** p < 0.001, and **** p < 0.0001. Data is expressed as mean of quadruplicates; error bars indicate standard deviation.*

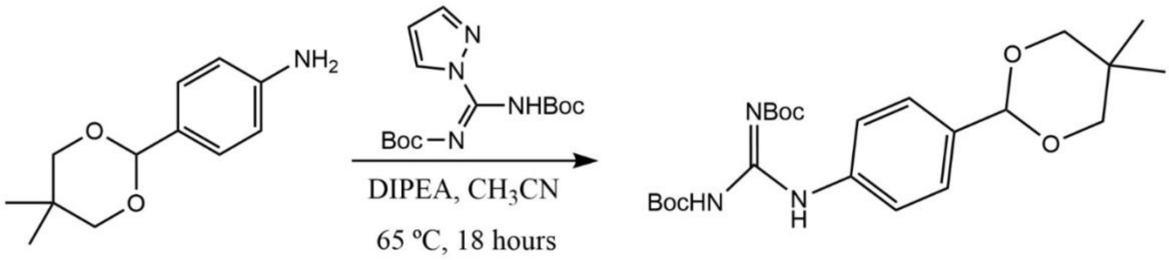

Figure S6. Synthesis of 1-(4-formylphenyl)guanidine) (T2). *Synthetic strategy for the synthesis of 1-(4-formylphenyl)guanidine) (T2).*

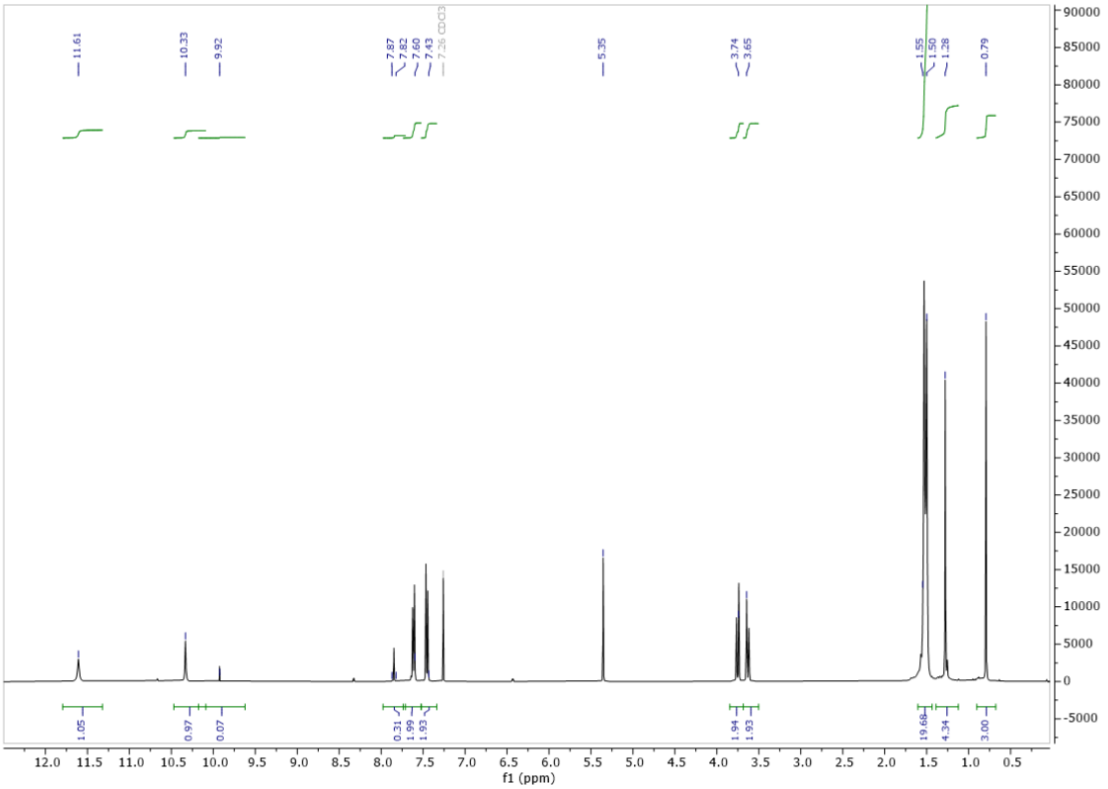

Figure S7. Synthesis of 1-(4-formylphenyl)guanidine) (T2). *Characterization of 1-(4-formylphenyl)guanidine) (T2) by H NMR (400 MHz, CDCl3).*

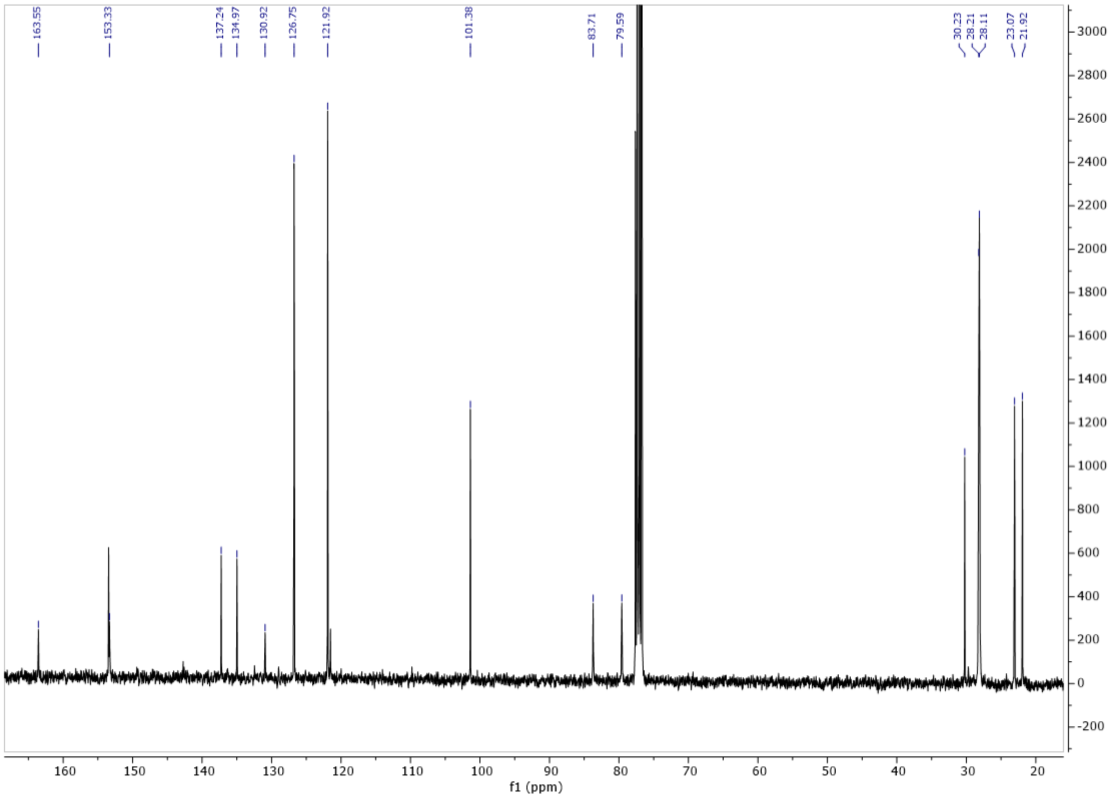

Figure S8. Synthesis of 1-(4-formylphenyl)guanidine) (T2). *Characterization of 1-(4-formylphenyl)guanidine) (T2) by C NMR (101 MHz, CDCl3).*

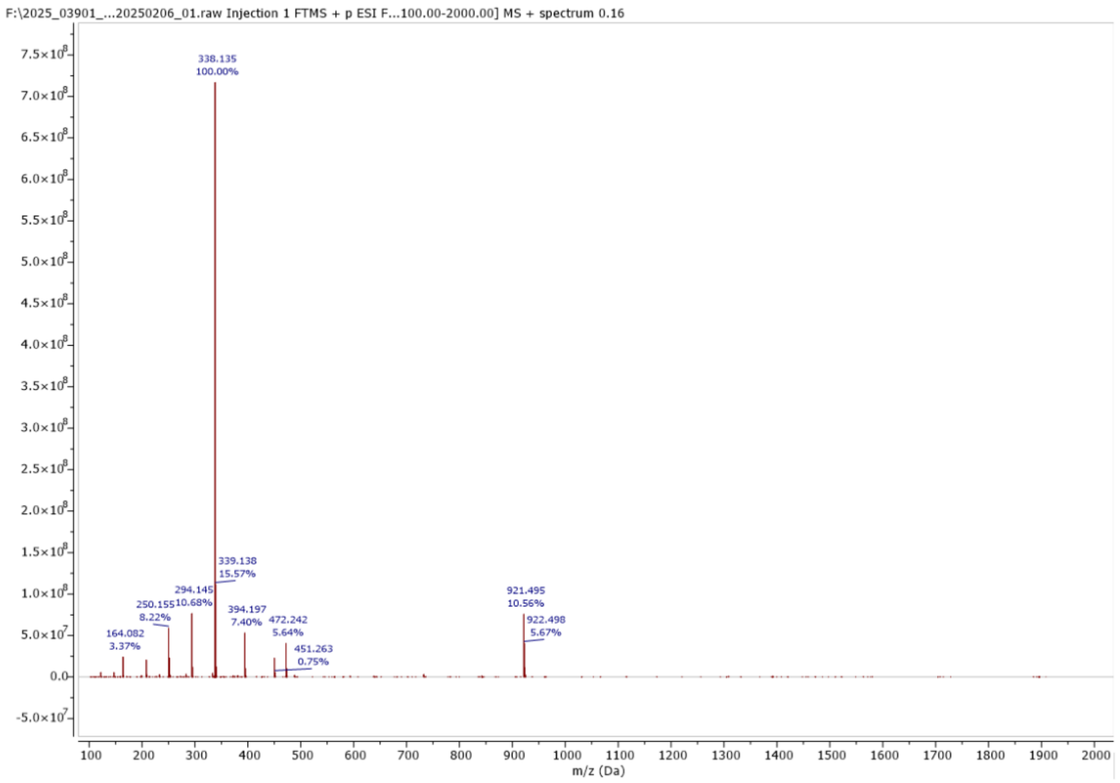

Figure S9. Synthesis of 1-(4-formylphenyl)guanidine) (T2). *Characterization of 1-(4-formylphenyl)guanidine) (T2) by ESI-MS (CH3Cl) m/z [2M+ Na]+ 921,495.*

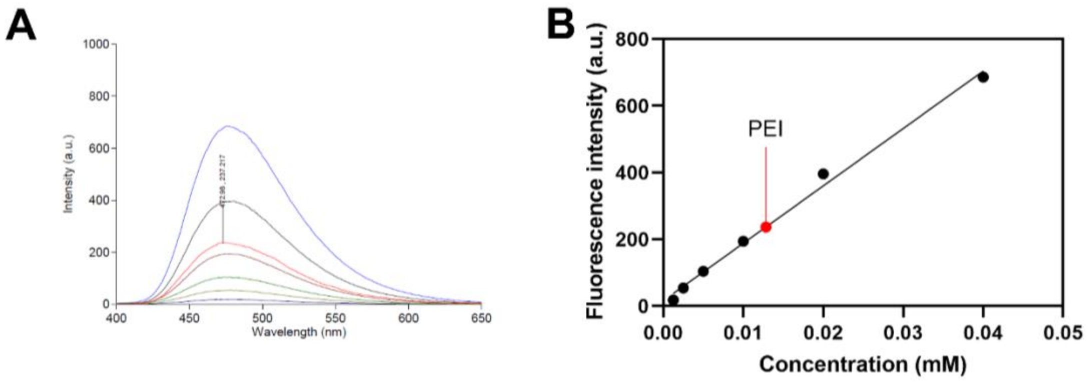

Figure S10. Estimation of unmodified PEI free amines. *Fluorescence intensity of PEI (Red line (A) or dot (B)) concerning ethanolamine (rest of the values) after functionalization of the free amines in both compounds with fluorescamine. Data is represented concerning wavelength (A) or concentration (B).*

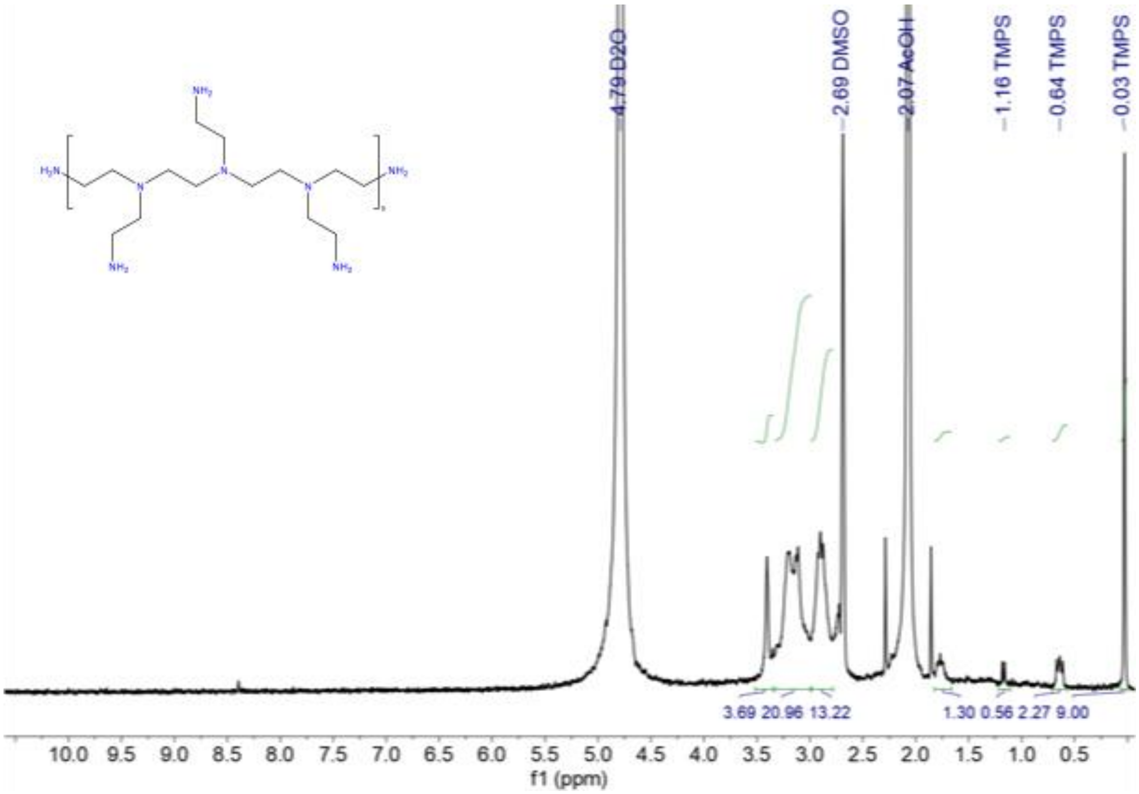

Figure S11. ***^{1}H NMR spectra of PEI*** *in acetate buffer (100 mM, pH 3.0) in D_2O. Sodium 3-(trimethylsilyl)propane-1-sulfonate (**TMPS**) used as an internal reference.*

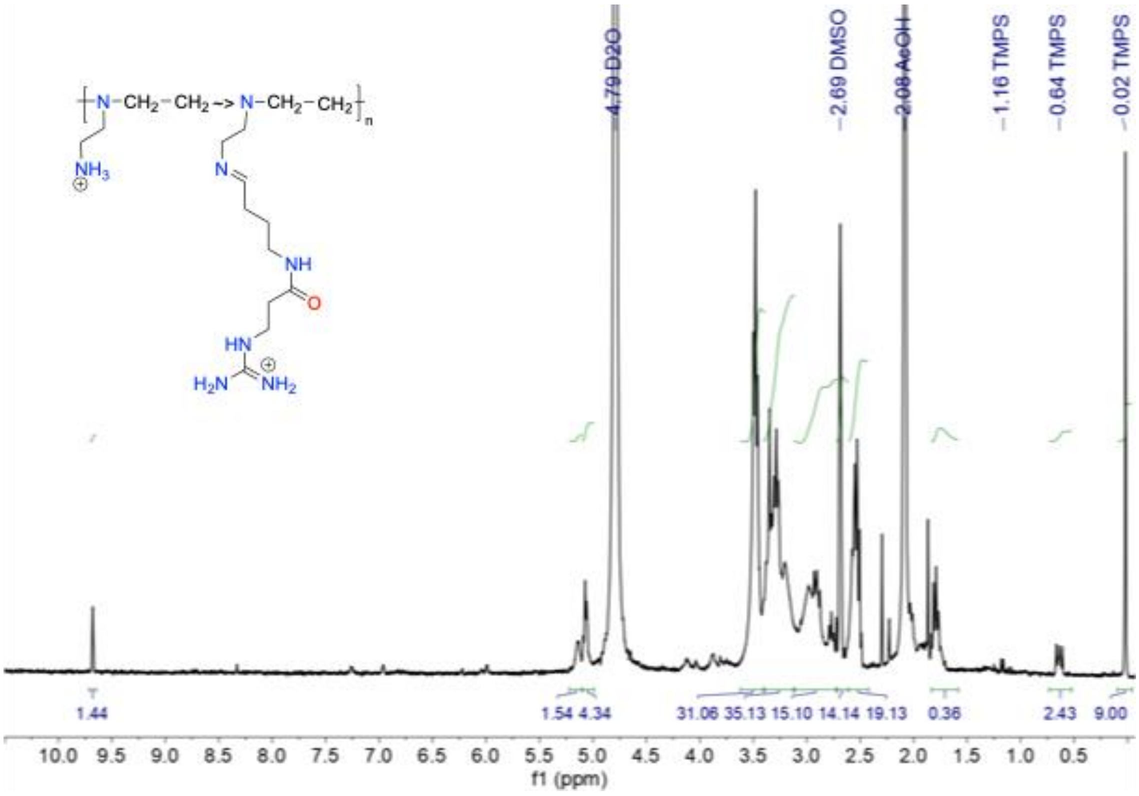

Figure S12. ***^{1}H NMR spectra of p[PEI-mod-T1$_{0.30}$] (PEIT1)*** *in acetate buffer (100 mM, pH 3.0) in D_2O. Sodium 3-(trimethylsilyl)propane-1-sulfonate (**TMPS**) used as an internal reference.*

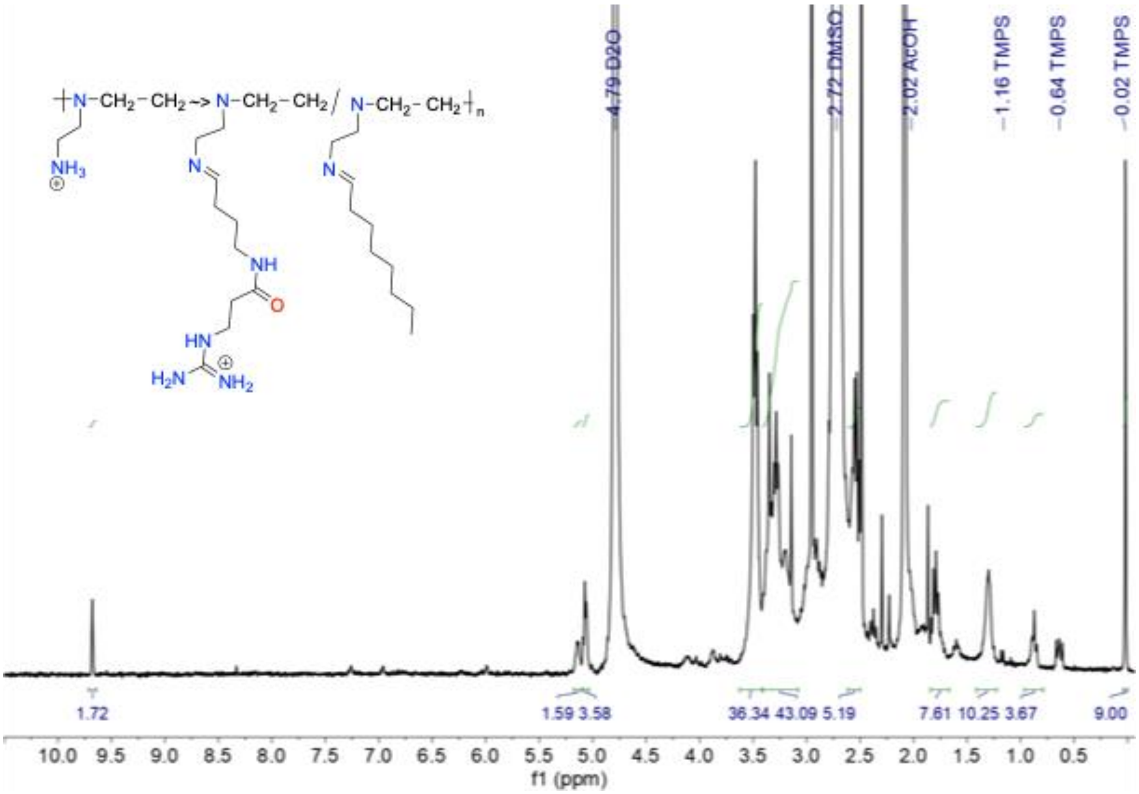

Figure S13. ^{1}H NMR spectra of p[PEI-mod-T1$_{0.32}$T3A$_{0.6}$] (PEIT1T3A) *in acetate buffer (100 mM, pH 3.0) in D_2O. Sodium 3-(trimethylsilyl)propane-1-sulfonate (**TMPS**) used as an internal reference.*

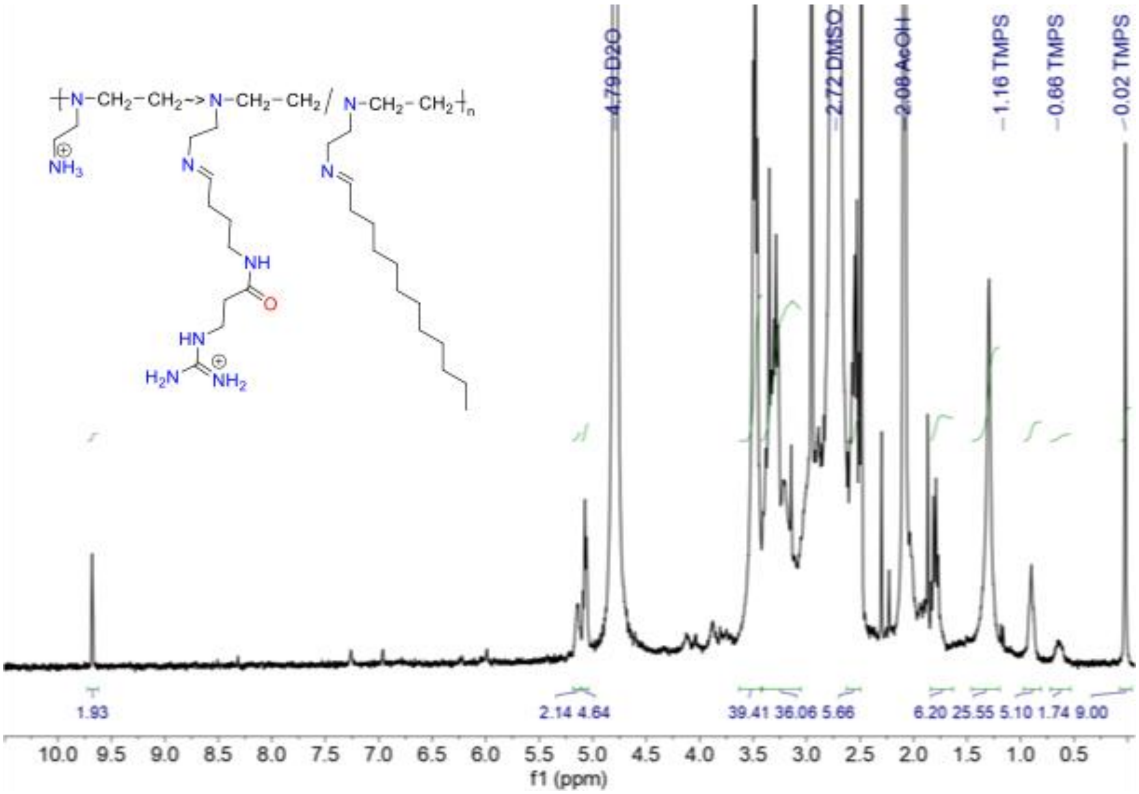

Figure S14. ^{1}H NMR spectra of p[PEI-mod-T1$_{0.34}$T3B$_{0.6}$] (PEIT1T3B) *in acetate buffer (100 mM, pH 3.0) in D_2O. Sodium 3-(trimethylsilyl)propane-1-sulfonate (**TMPS**) used as an internal reference.*

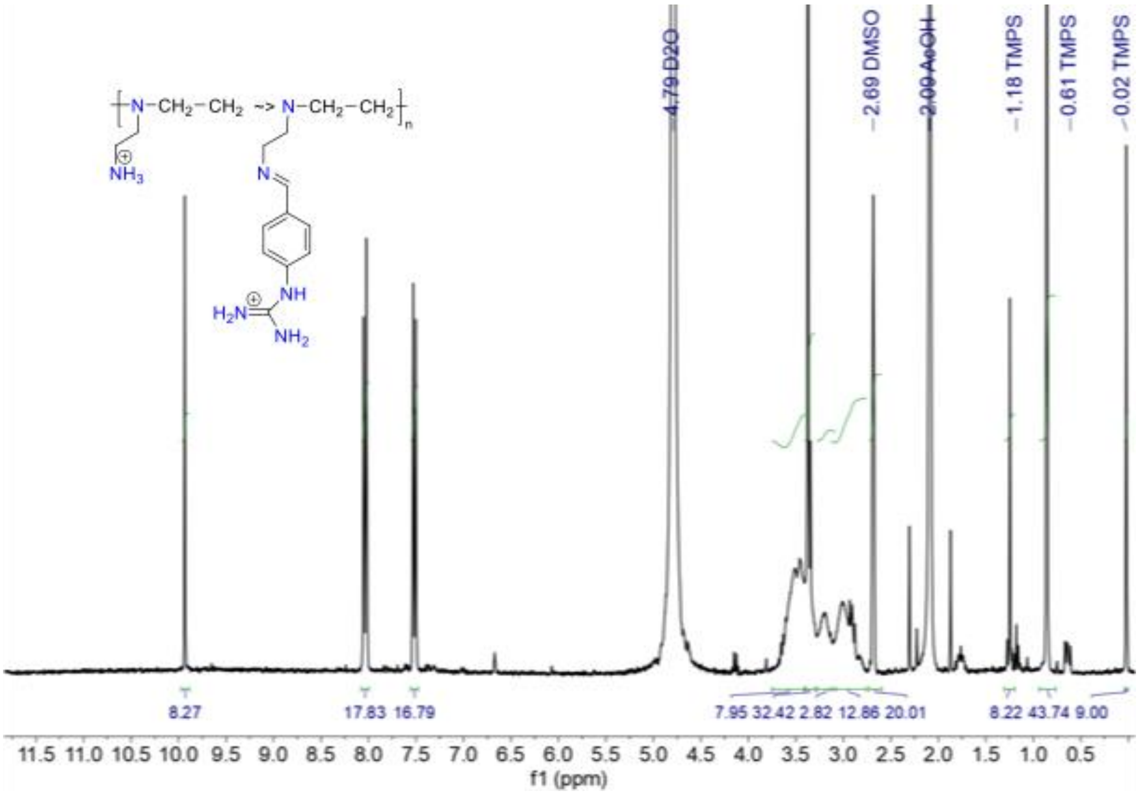

Figure S15. ***^{1}H NMR spectra of p[PEI-mod-T2$_{0.19}$] (PEIT2)*** *in acetate buffer (100 mM, pH 3.0) in D_2O. Sodium 3-(trimethylsilyl)propane-1-sulfonate (**TMPS**) used as an internal reference.*

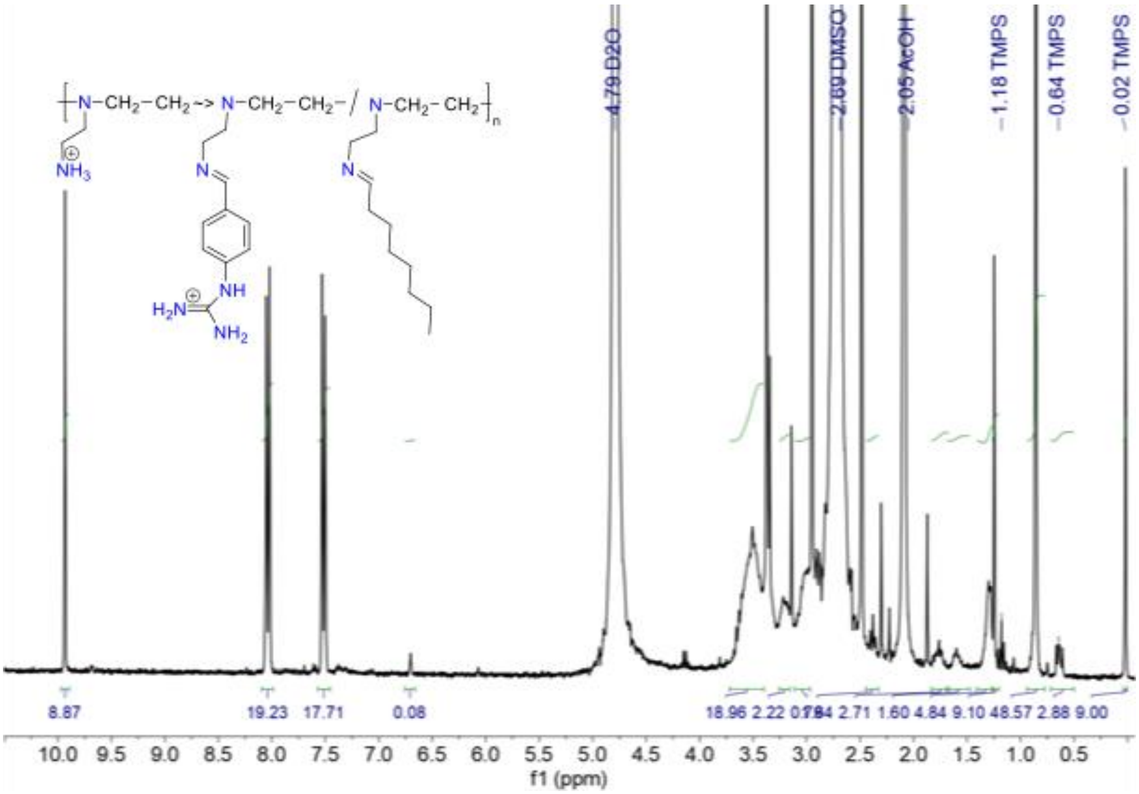

Figure S16. ***^{1}H NMR spectra of p[PEI-mod-T2$_{0.15}$T3A$_{0.60}$] (PEIT2T3A)*** *in acetate buffer (100 mM, pH 3.0) in D_2O. Sodium 3-(trimethylsilyl)propane-1-sulfonate (**TMPS**) used as an internal reference.*

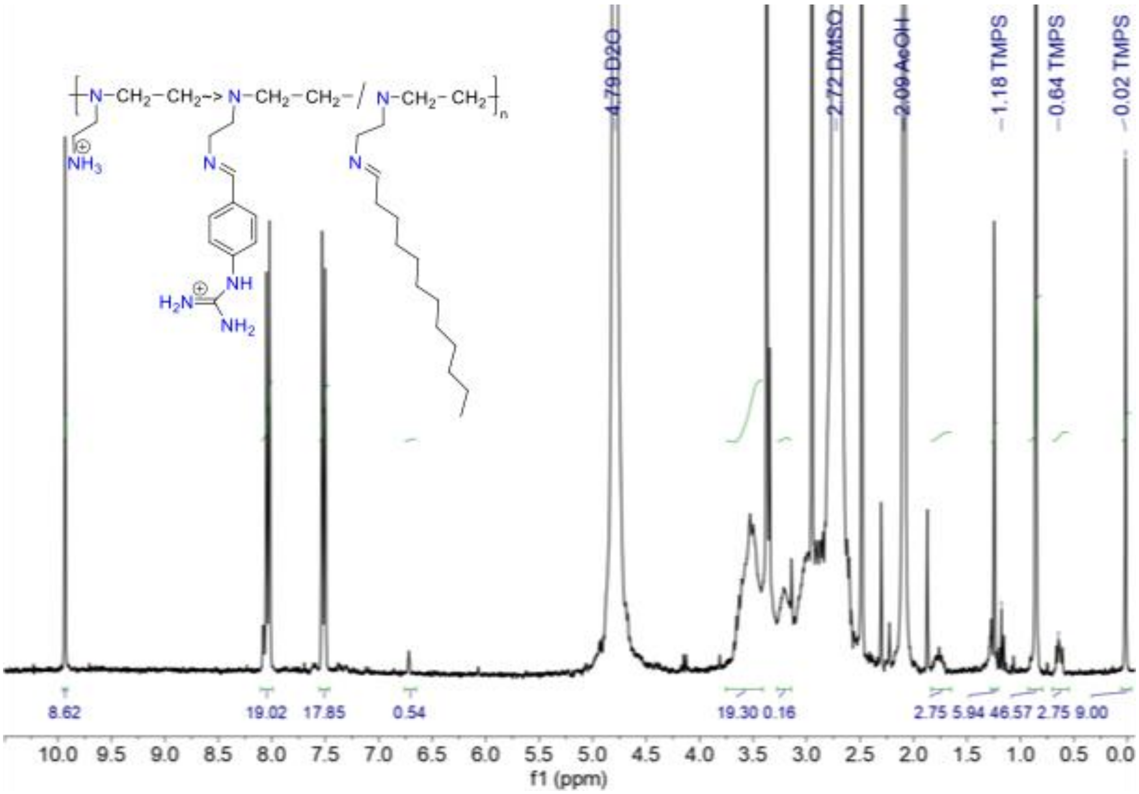

Figure S17. ***^{1}H NMR spectra of p[PEI-mod-T2$_{0.17}$T3B$_{0.60}$] (PEIT2T3B)*** *in acetate buffer (100 mM, pH 3.0) in D_2O. Sodium 3-(trimethylsilyl)propane-1-sulfonate (**TMPS**) used as an internal reference.*

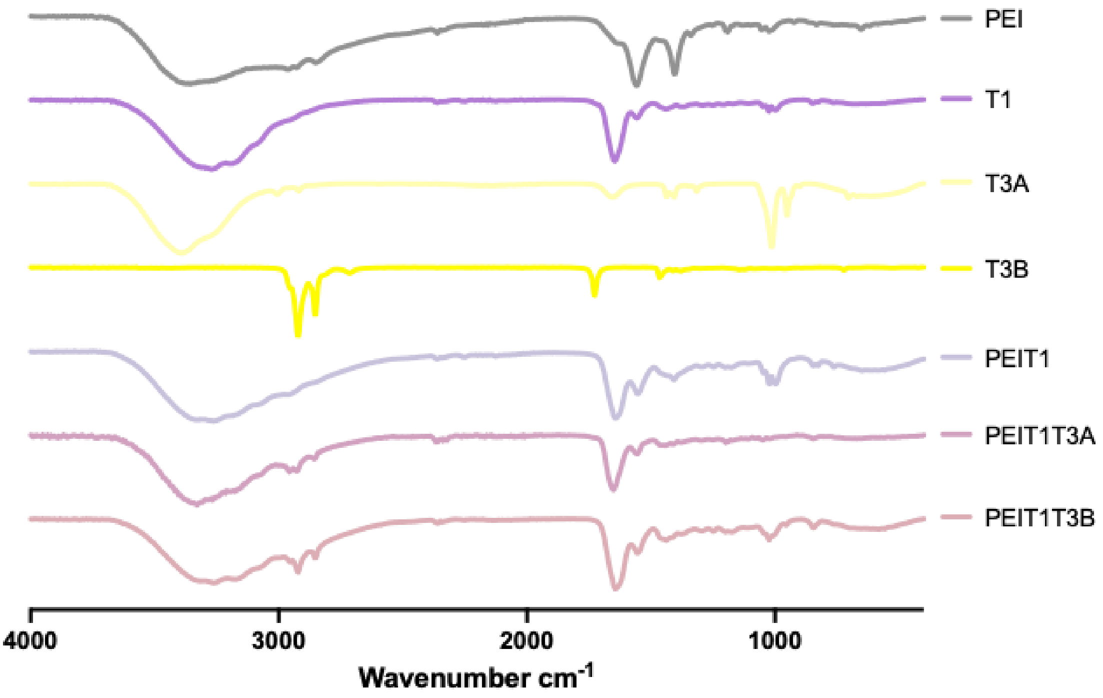

Figure S18. IR spectra of PEI and its functionalization with T1 or T1/T3 mixtures*. T1, T3A and T3B shown for comparison.*

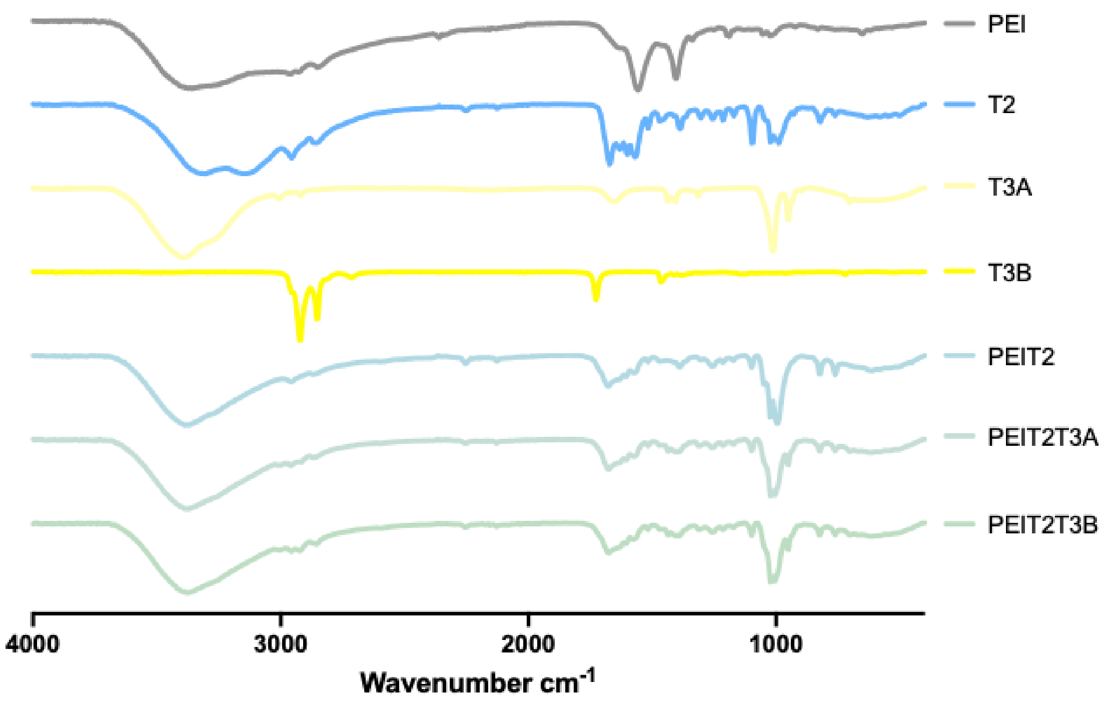

Figure S19. IR spectra of PEI and its functionalization with T2 or T2/T3 mixtures. *T2, T3A and T3B shown for comparison.*
